# Collagen Type III Alpha 1 chain regulated by GATA‐Binding Protein 6 affects Type II IFN response and propanoate metabolism in the recurrence of lower grade glioma

**DOI:** 10.1111/jcmm.15705

**Published:** 2020-08-05

**Authors:** Runzhi Huang, Zhenyu Li, Xiaolong Zhu, Penghui Yan, Dianwen Song, Huabin Yin, Peng Hu, Ruoyi Lin, Shengyu Wu, Tong Meng, Jie Zhang, Zongqiang Huang

**Affiliations:** ^1^ Department of Orthopedics The First Affiliated Hospital of Zhengzhou University Zhengzhou China; ^2^ Division of Spine Department of Orthopedics Tongji Hospital affiliated to Tongji University School of Medicine Shanghai China; ^3^ Tongji University School of Medicine Shanghai China; ^4^ Department of Orthopedics Shanghai General Hospital School of Medicine Shanghai Jiaotong University Shanghai China; ^5^ Shanghai First Maternity and Infant Hospital Tongji University School of Medicine Shanghai China

**Keywords:** low‐grade glioma, prognosis, propanoate metabolism, recurrence, type II IFN response

## Abstract

Some studies suggested the prognosis value of immune gene in lower grade glioma (LGG). Recurrence in LGG is a tough clinical problem for many LGG patients. Therefore, prognosis biomarker is required. Multivariate prognosis Cox model was constructed and then calculated the risk score. And differential expressed transcription factors (TFs) and differential expressed immune genes (DEIGs) were co‐analysed. Besides, significant immune cells/pathways were identified by single sample gene set enrichment analysis (ssGSEA). Moreover, gene set variation analysis (GSVA) and univariate Cox regression were applied to filter prognostic signalling pathways. Additionally, significant DEIG and immune cells/pathways, and significant DEIG and pathways were co‐analysed. Further, differential enriched pathways were identified by GSEA. In sum, a scientific hypothesis for recurrence LGG including TF, immune gene and immune cell/pathway was established. In our study, a total of 536 primary LGG samples, 2,498 immune genes and 318 TFs were acquired. Based on edgeR method, 2,164 DEGs, 2,498 DEIGs and 31 differentials expressed TFs were identified. A total of 106 DEIGs were integrated into multivariate prognostic model. Additionally, the AUC of the ROC curve was 0.860, and *P* value of Kaplan‐Meier curve < 0.001. GATA6 (TF) and COL3A1 (DEIG) were selected (*R* = 0.900, *P* < 0.001, positive) as significant TF‐immune gene links. Type II IFN response (*P* < 0.001) was the significant immune pathway. Propanoate metabolism (*P* < 0.001) was the significant KEGG pathway. We proposed that COL3A1 was positively regulated by GATA6, and by effecting type II IFN response and propanoate metabolism, COL3A1 involved in LGG recurrence.

## 1 INTRODUCTION

Immune genes involve in tumour genesis, development and prognosis,^1^, ^2^ and these genes can be applied in mechanism exploring, diagnosis, therapy target identification and prognosis.^2^, ^3^ Some studies indicated that immune‐related lncRNA showed its prognosis value in lower grade glioma (LGG).^2^

LGG is a serial common centre nervous system tumour. According to classification of WHO, glioma can be divided into Ⅰ, Ⅱ, Ⅲ and Ⅳ grade, and grade Ⅰ and Ⅱ are included in LGG. And estimated by the American Cancer Society, it will be 23,890 new cases and 18,020 death in America.^4^ Generally, the prognosis of primary LGG is better than higher grade glioma, and the median overall survival is 5‐10 years.^5^ However, the recurrence, progression and deterioration make the therapy tough. And the recurrence rates of grade Ⅰ and Ⅱ are 84.5%^6^ and 57.6%,^6^ respectively. Besides, 50%‐75% LGG patients died of progression and deterioration.^7^ Therefore, for optimizing treatment, the biomarker for recurrence is required in the early stage. And we proposed immune gene might participate in LGG recurrence.

In our study, differential expressed immune genes (DEIGs) were identified, and univariate Cox regression was utilized to access the prognostic value. Additionally, significant prognosis DEIGs were integrated into multivariate prognosis Cox model and then calculated the risk score. Univariate and multivariate Cox regression were applied to validate risk score as the independent prognosis factor. Similarly, differential expressed transcription factors (TFs) were identified. Then, co‐analysis for differential expressed TFs and DEIGs to identify the significant TF‐DEIG relationship was performed. Based on CIBERSORT, immune cells/pathways were identified, and significant immune cells/pathways were identified by ssGSEA. And significant DEIG and immune cells/pathways to identify the significant immune cell/pathway were co‐analysed. Moreover, GSVA was utilized to identify the expression level in clinical LGG sample, and univariate Cox regression evaluated the prognosis value of Kyoto Encyclopedia of Genes and Genomes (KEGG) pathways. Further, differential expressed KEGG pathways were identified by GSEA. Additionally, significant DEIG and KEGG pathways to identify the significant KEGG pathway were co‐analysed. In sum, a scientific hypothesis for LGG recurrence based on TF, immune gene, immune cell/pathway and KEGG pathway was established.

## 2 MATERIAL AND METHOD

### 2.1 Data acquisition

Based on TCGA database (https://tcga‐data.nci.nih.gov), gene expression profiles and clinical information of 536 primary LGG samples were acquired. Data of 2498 immune genes were available from ImmPort (http://www.immport.org/) and MsigDB (http://software.broadinstitute.org/gsea/msigdb/) database. Besides, data of 318 TFs were downloaded from Cistrome database (http://cistrome.org).

### 2.2 Differential expressed genes identification

Gene expression level of clinical samples in TCGA database was performed by edgeR method, differential expressed genes (DEGs) based on whether recurrence or not were identified by log2 FC (Fold charge) and false discovery rate (FDR) *P* value. When log2 FC >1.0 or <−1.0, and FDR *P* value < 0.05, DEGs were defined. Further, Gene Oncology (GO) and Kyoto Encyclopedia of Genes and Genomes (KEGG) enrichment analysis were utilized to explore clinical significance of DEGs. Similarly, immune genes were performed by edgeR method, when log2 FC >1.0 or <−1.0, and FDR *P* value < 0.05, DEIGs were defined. In addition, to access the prognostic value, DEIGs were screened by univariate Cox regression. Also, TFs were screened by edgeR method, differential expressed TFs were identified as log2 >1.0 or <−1.0, and FDR *P* value < 0.05.

### 2.3 Multivariate model construction and independent prognosis analysis

To predict the prognosis of LGG, differential expressed prognostic *s*ignificance immune genes were integrated into multivariate model. Additionally, the accuracy of the model was tested by receiver operator characteristic (ROC) curve. And for each LGG sample, risk score was calculated by this following formula:

RiskScore=β1×DEIG1+β2×DEIG2+β3×DEIG3+⋯+βn×DEIGn.

For each single sample, ‘n’ represented the number of DEIG in the multivariate model, ‘β’ represented regression coefficient of each DEIG, and DEIGn represented the expression level of the Nth DEIG in the corresponding sample. Then, median of risk score was selected to distinguish the high and low‐risk score, and samples were divided into the high‐ and low‐risk group, respectively. Besides, Kaplan‐Meier curve was utilized to predict the prognosis value of the multivariate model based on risk score.

Further, the value of independently prognostic for risk score was evaluated by univariate and multivariate Cox regression analysis. And other demographic information like age, gender and race was applied for correction.

### 2.4 Regulatory network for immune genes and TFs

To validate the clinical relationship with recurrence/ new tumour event, non‐parametric test for DEIGs was utilized. The Pearson correlation analysis was utilized to co‐analyse differential TFs and DEIGs. When correlation coefficient more than 0.300 and *P* value < 0.001, the corresponding regulatory relationships based on TFs and DEIGs were considered to be statistically significant. Besides, the most significant regulatory relationship was selected, and the most significant TF and DEIG were selected, respectively.

### 2.5 Co‐analysis the relationship of immune gene with immune cells/pathways and KEGG pathway

To identify the characteristic of immune cells and pathways in the downstream of DEIGs in clinical samples, single sample gene set enrichment analysis (ssGSEA) was utilized to filter significant immune cells and pathways. Besides, the Pearson analysis was used to identify the correlation between the most significant DEIG and immune cells/pathways. When *P* value < 0.05, the interplay was regarded as significant. And the most significant immune cell or pathway was decided by the largest correlation coefficient.

Aiming to filter prognostic KEGG pathway, gene set variation analysis (GSVA) was applied to access the expression level of KEGG pathways, and all KEGG pathways were in the downstream of the most significant DEIG. In addition, univariate Cox regression was utilized to calculate the prognostic value of KEGG pathways. And survival‐related KEGG pathways were identified. What's more, to co‐analyse the most significant DEIG and survival‐related KEGG pathways, the Pearson analysis was utilized, and *P* value < 0.05 was defined as significant.

### 2.6 Regulatory network for TFs, immune genes, immune cell and KEGG pathways

Gene set enrichment analysis (GSEA) was utilized to identify significant KEGG pathway between primary and recurrence LGG samples in clinical data. According to the result of GSVA and GSEA, the most significant survival‐related and recurrent‐related KEGG pathway was selected.

The regulatory network was constructed based on the interaction of the most significant TF, DEIG, top 9 immune cells/pathways and top three KEGG pathways.

### 2.7 Multidimensional validation

To validate our scientific hypothesis and decrease inherent deficient in silico, multidimensional validation was applied based on the following online databases. Immune gene set was utilized to find related genes of corresponding immune cells/pathways.^8^ Besides, to find top 5 genes represent KEGG pathway, Pathway Card (https://pathcards.genecards.org/) was applied. Gene Expression Profiling Interactive Analysis (GEPIA),^9^ Oncomine,^10^ PROGgeneV2,^11^ UALCAN,^12^ Linkedomics,^13^ cBioportal,^14^ Genotype‐Tissue Expression (GTEx),^15^ UCSC xena,^16^ Cancer Cell Line Encyclopedia (CCLE),^17^ Expression atlas,^18^ The Human Protein Altas,^19^ CGGA^20^ and String^21^ were utilized to validate the hypothesis.

### 2.8 Statistics analysis

For all analysis process, tow‐sided *P* value < 0.05 was defined as statistically significant. And R version 3.6.1 was utilized for analysis in silico (Institute for Statistics and Mathematics, Vienna, Austria; www.r‐project.org) (Package: e1071, parallel, preprocessCore, sva, limma, edgeR, ggplot2, survminer, survival, rms, randomForest, pROC, glmnet, pheatmap, timeROC, vioplot, corrplot, ConsensusClusterPlus, forestplot, survivalROC, beeswarm, chromVAR, Biostrings, BSgenome.Hsapiens.UCSC.hg38, TxDb.Hsapiens.UCSC.hg38.knownGene, clusterProfiler, org.Hs.eg.db, ggplot2, karyoploteR, limma, pheatmap, TCGAbiolinks, dplyr, kableExtra, ggplot2, ggrepel, ggthemes, gridExtra, msigdbr, data.table, GSVA and stringr).

## 3 RESULT

### 3.1 Differential immune gene identification

**FIGURE 1:**
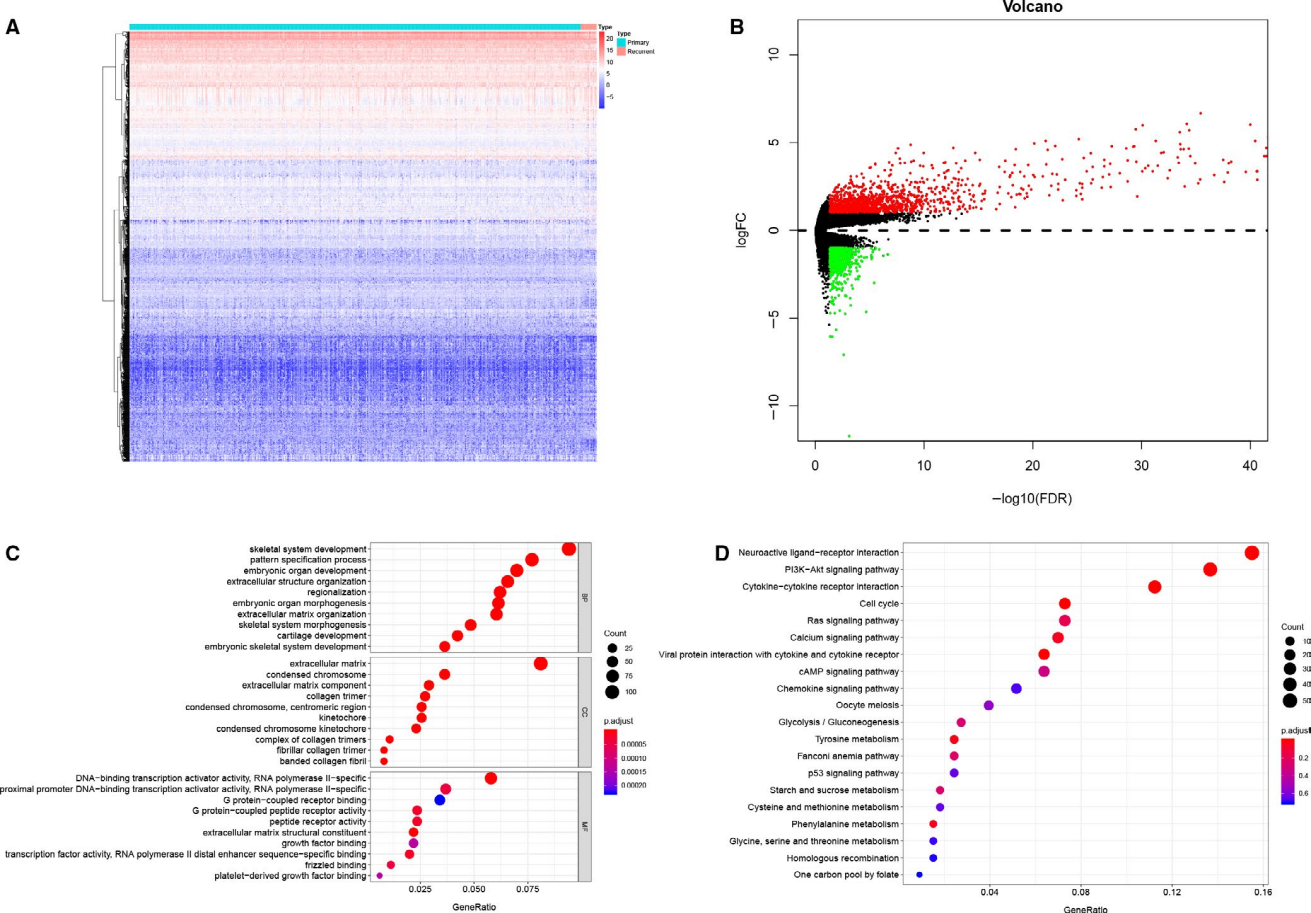
Heatmap for genes from samples (A), blue and red represent primary and recurrent samples. Volcano plot for genes from samples (B), green and red dots represent significant differential expression. Bubble plot for GO (C) and KEGG (D) enrichment analysis. GO, Gene Oncology; KEGG, Kyoto Encyclopedia of Genes and Genomes

**FIGURE 2:**
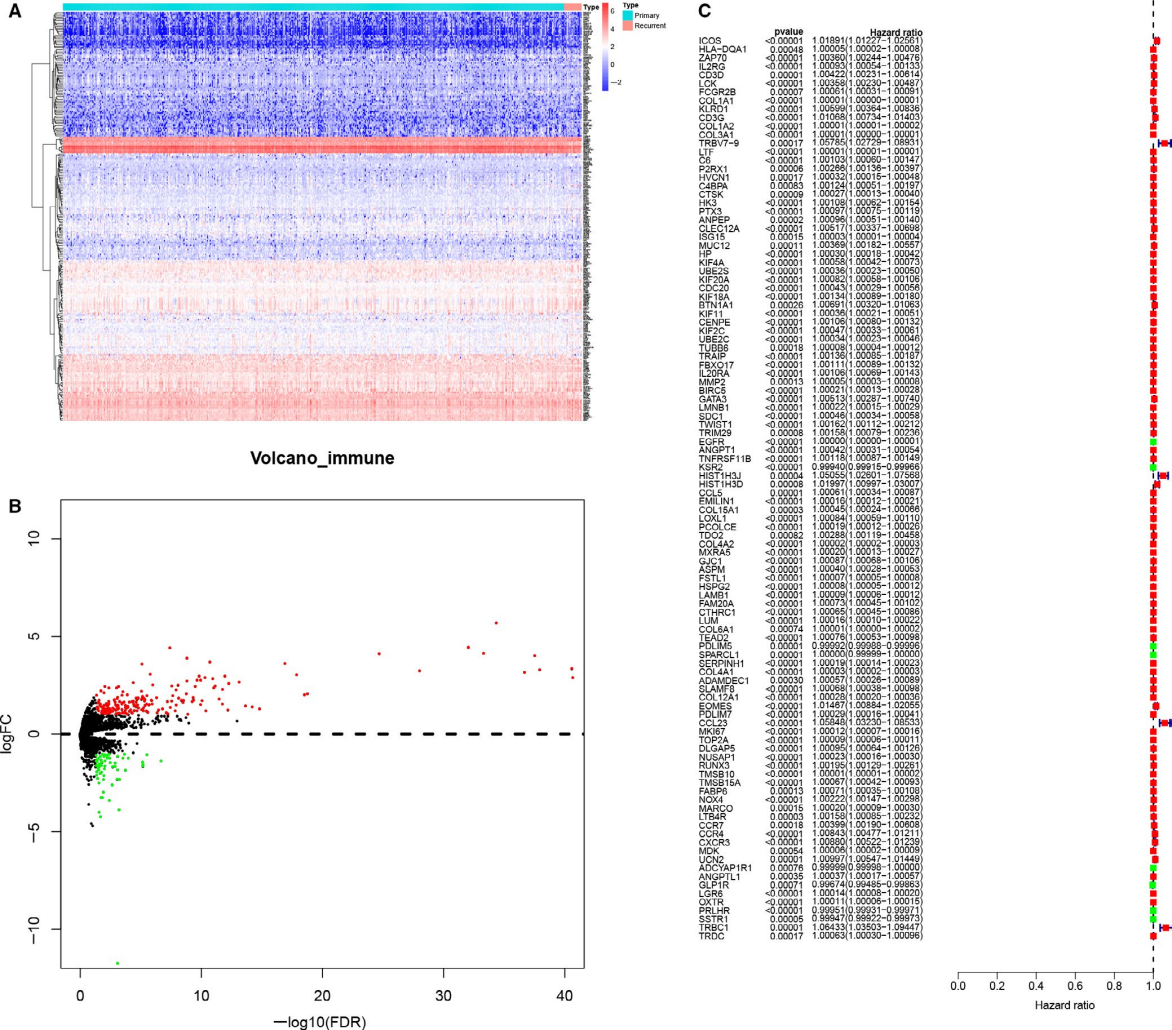
Heatmap for immune genes (A), blue and red represent primary and recurrent samples. Volcano plot for immune genes (B), green and red dots represent significant differential expression. Univariate Cox regression for immune genes (C)

All analysis process was illustrated in Figure S1. And all baseline information of LGG samples were summarized in Table 1. Gene expression level from 536 LGG samples in TCGA database was analysed, log2 FC >1 or <−1, and *P* value < 0.05 was defined as expressed significantly and differentially. Heatmap plot (Figure 1A) and volcano plot (Figure 2B) demonstrated a total of 2,164 DEGs based on primary and recurrence were identified. Additionally, GO (Figure 1C) and KEGG (Figure 1D) enrichment analyses were performed for exploring the clinical link of DEGs. Most significant GO for BP, CC and MF was skeletal system development, extracellular matrix and DNA‐binding transcription activator activity, RNA polymerase Ⅱ‐specific; most significant KEGG pathway was neuroactive ligand‐receptor interaction. Moreover, based on edgeR method for 2498 immune genes, when log2 FC >1 or <−1 and *P* value < 0.05, DEIGs were defined. And heatmap plot (Figure 2A) and volcano plot (Figure 2B) showed 269 DEIGs included in 2164 DEGs. In addition, to evaluate the prognosis value of DEIGs, univariate Cox regression was utilized and 99 DEIGs were significant as risk factor and 7 DEIGs were significant as protective factor (Figure 2C).

**TABLE 1:** Baseline information of 536 patients diagnosed with lower grade glioma

Variables	Total patients (N = 536)
Age, years	
Mean ± SD	42.67 ± 13.32
Median (Range)	40.00 (14‐87)
Gender	
Female	242 (45.15%)
Male	293 (54.66%)
NA	1 (0.19%)
Race	
White	494 (92.16%)
Black or African‐American	22 (4.11%)
American‐Indian or Alaska native	1 (0.19%)
Asian	8 (1.49%)
NA	11 (2.05%)
Sample type	
Primary tumour	516 (96.27%)
Recurrent tumour	20 (3.73%)
New tumour event	
Yes	148 (27.61%)
No	279 (52.05%)
NA	109 (20.34%)

Abbreviation: NA, not available; SD, Standard deviation.

### 3.2 Multivariate prognostic model and independent prognostic factor identification

**FIGURE 3:**
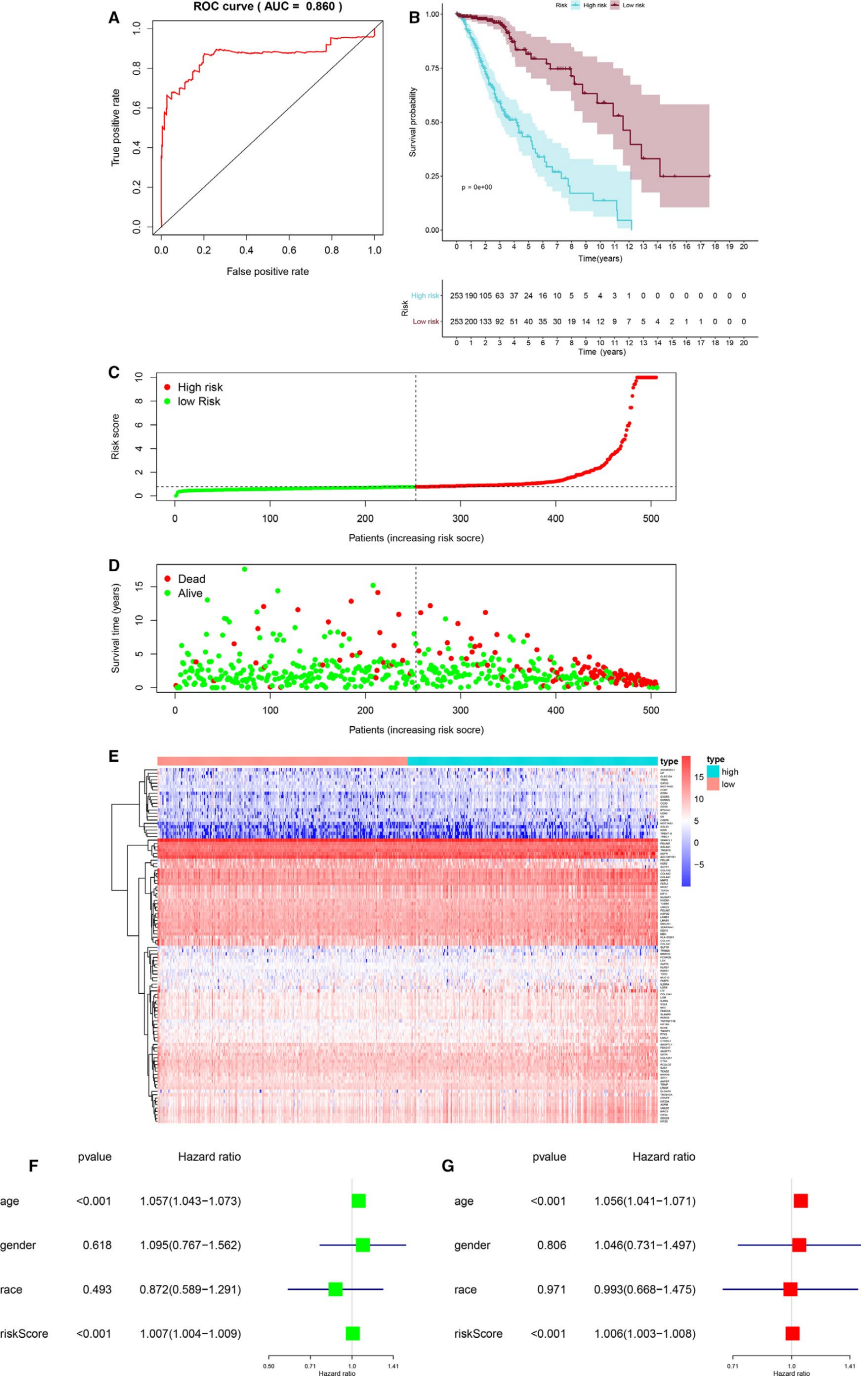
ROC curve for (AUC = 0.860) prognostic immune genes (A). Overall survival Kaplan‐Meier curve for prognostic immune genes (B). Scatterplot (C) for the high and low‐risk group samples, red and green represent high‐risk and low‐risk groups. Risk plot (D) for dead and alive samples, red and green represent dead and alive samples. Heatmap for prognostic immune genes (E), red and green represent low‐risk and high‐risk groups. Univariate (F) and multivariate (G) Cox regression model. Green and red represent univariate and multivariate Cox regression model. AUC, area under the curve; ROC, The receiver operator characteristic

A total of 106 DEIGs were integrated into multivariate prognostic model to access the prognosis of LGG, and ROC curve (Figure 3A) was applied to evaluate the efficiency of the multivariate model (AUC = 0.860). Besides, to validate the prognostic value of the risk score, Kaplan‐Meier curve (Figure 3B) was utilized, and *P* value < 0.001. Additionally, scatter plot (Figure 3C) and risk curve (Figure 3D) were also demonstrated the clinical link of risk score based on clinical samples. Moreover, in the heatmap plot (Figure 3E), green and red represented the high‐ and low‐risk group samples, and 21 DEIGs lower than low‐risk group, 32 DEIGs higher than the high‐risk group and 53 DEGs were not significant.

To explore whether risk score was an independent prognostic factor or not, univariate (HR = 1.007, 95% CI (1.004‐1.009),* P* < 0.001) (Figure 3F) and multivariate (HR = 1.006, 95% CI (1.003‐1.008), *P* < 0.001) (Figure 3G) Cox regression models were utilized. Therefore, risk score was defined as an independent prognostic factor.

### 3.3 Co‐analysis DEIGs and TFs

**FIGURE 4:**
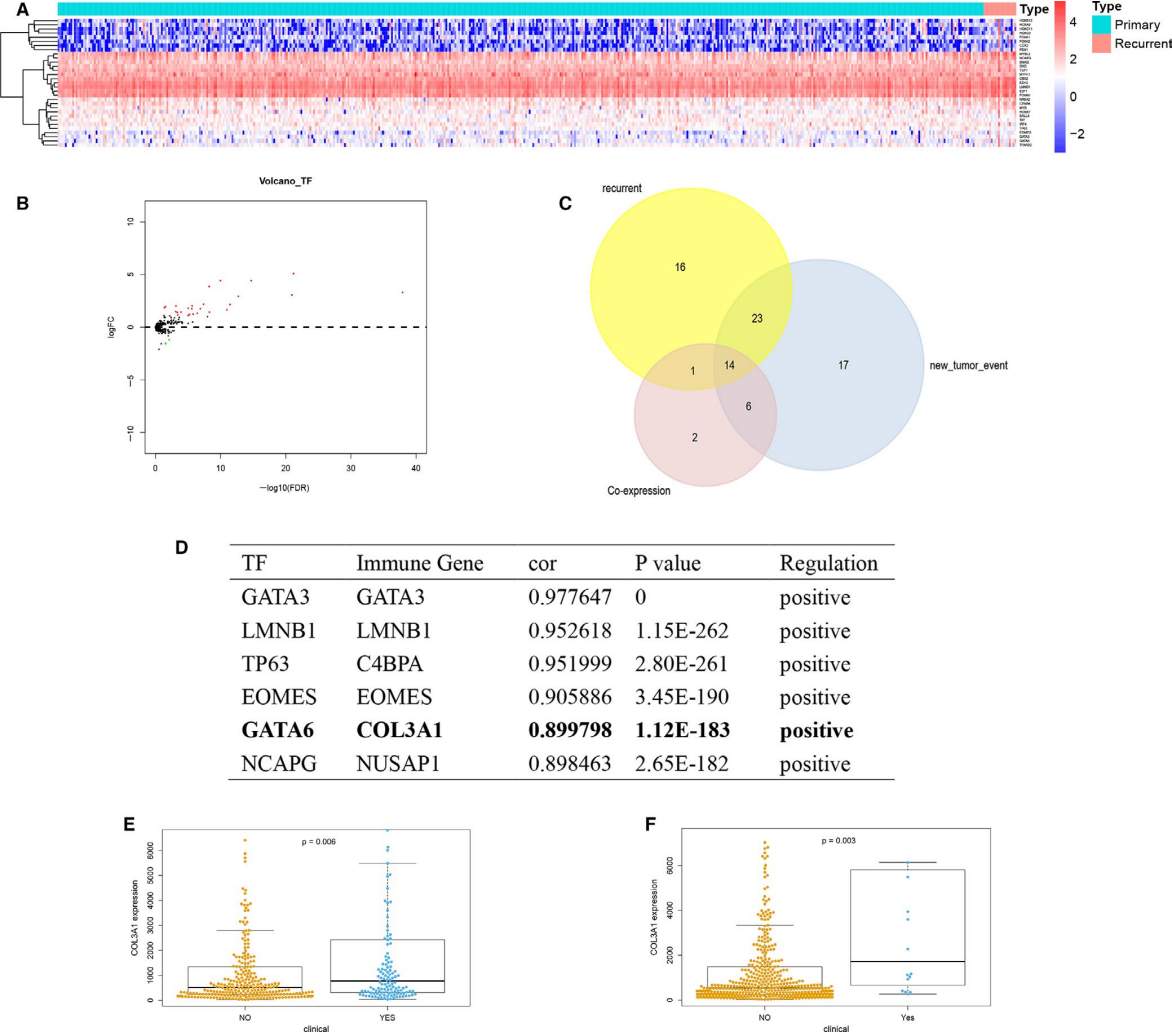
Heatmap for TFs (A), blue and red represent primary and recurrent samples. Volcano plot for TFs (B), green and red dots represent significant differential expression. Venn plot for co‐expressed, recurrent and new tumour event immune genes (C). Result for co‐analysis immune genes and TFs (D). Non‐parametric test beeswarm plot for new tumour event (E) and recurrent (F) and COL3A1. TF, transcription factor

EdgeR method for identified differential expressed TFs, and log2 FC >1 or <−1 and *P* value < 0.05 was set. Heatmap (Figure 4A) and volcano (Figure 4B) plots illustrated 31 differential expressed TFs from 318 TFs. Then, co‐analysed differential expressed TFs and DEIGs, links of 11 TFs and 23 DEIGs were significant. Further, to identify DEIGs related to clinical and TFs, the Venn plot (Figure 4C) of recurrent (54 immunegenes), TFs‐related (23 immune gens) and new tumour event (60 immune genes) was applied, and 14 DEIGs were selected. Besides, the Pearson analysis results (Figure 4D) for regulatory relationship of TFs and immune genes, and the link of GATA6 (TF) and COL3A1 (DEIG) was selected (*R* = 0.900, *P* < 0.001, positive). What's more, Beeswam plots for new tumour event (*P* = 0.006) (Figure 4E) and recurrent (*P* = 0.003) (Figure 4F) showed the clinical relationship of COL3A1.

### 3.4 Co‐analysis DEIG with immune cells/pathways and KEGG pathways

**FIGURE 5:**
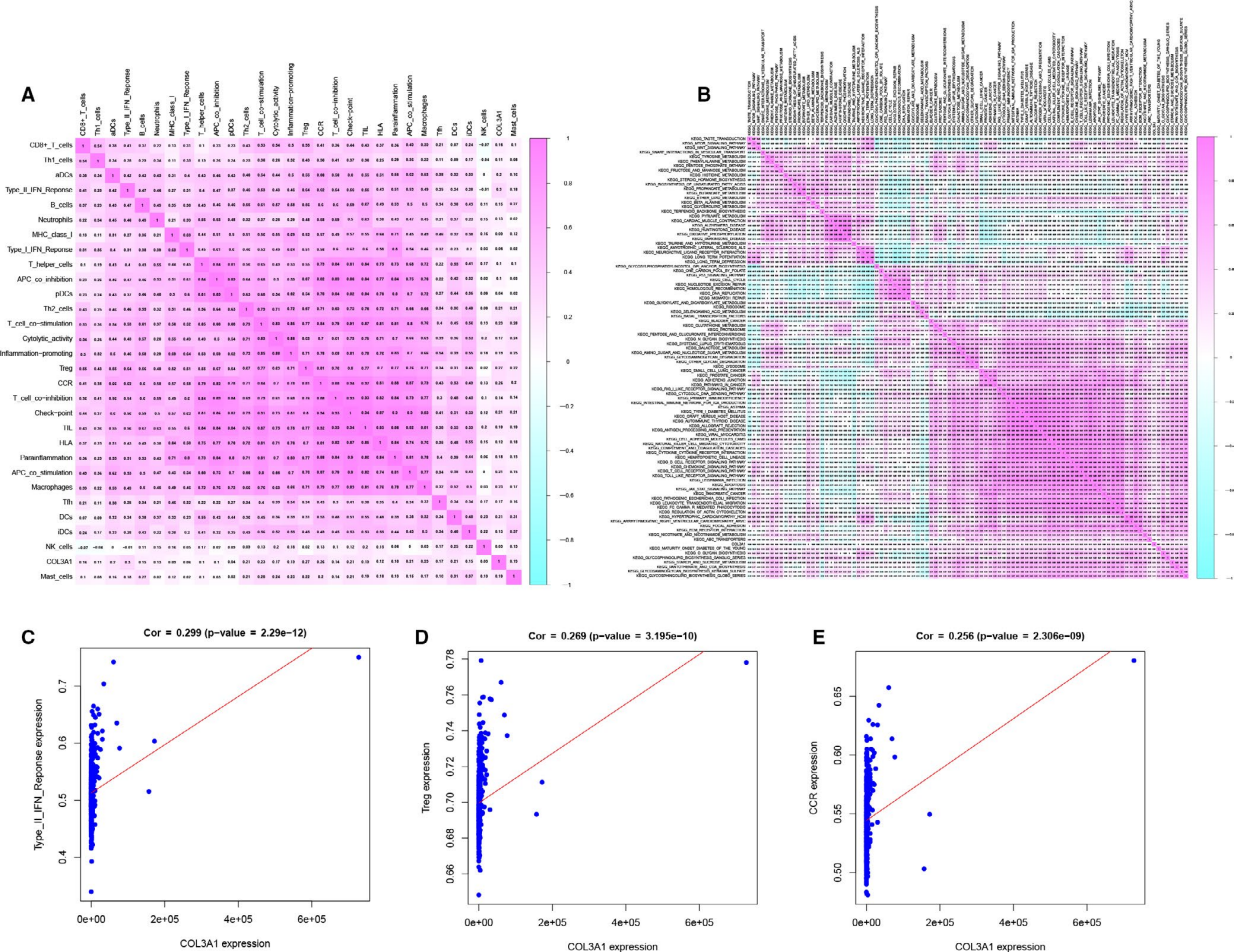
Co‐analysis immune cells/pathways and COL3A1 by ssGSEA analysis (A), co‐analysis KEGG pathways and COL3A1 by GSVA analysis (B). Correlation analysis for Type II IFN Reponse and COL3A1 (C), correlation analysis for Treg cell and COL3A1 (D), correlation analysis for CCR expression and COL3A1 (E) by ssGSEA analysis. GSVA, Gene Set Variation Analysis; ssGSEA, Single Sample Gene Set Enrichment Analysis

Based on CIBERSORT for LGG samples, 29 types immune cells or pathways were defined. Further, ssGSEA was utilized to find significant immune cells and pathways in overall survival, and 4 immune cells and 5 immune pathways were significant. Then, co‐analysed COL3A1 and 29 types immune cells or pathways, heatmap plot (Figure 5A) demonstrated the top three immune cells or pathways: type II IFN response (*R* = 0.300, *P* < 0.001), Treg (*R* = 0.270, *P* < 0.001) and CCR (*R* = 0.260, *P* < 0.001). The detail correlation of COL3A1 and type II IFN response (*P* < 0.001) (Figure 5C), Treg (*P* < 0.001) (Figure 5D) and CCR (*P* < 0.001) (Figure 5E) based on ssGSEA also showed. Eventually, type II IFN response was selected.

To find overall survival‐related KEGG pathways, 58 significant KEGG pathways were filtered by GSVA based on 184 KEGG pathways. And co‐analysed COL3A1 and 184 KEGG pathways, heatmap plot (Figure 5B) showed the top four KEGG pathways were ECM receptor interaction (*R* = 0.21, *P* < 0.001), N‐glycan biosynthesis (*R* = 0.19, *P* < 0.001), small cell lung cancer (*R* = 0.18, *P* < 0.001) and glycosaminoglycan degradation (*R* = 0.18, *P* < 0.001).

### 3.5 Significant KEGG pathway identification

**FIGURE 6:**
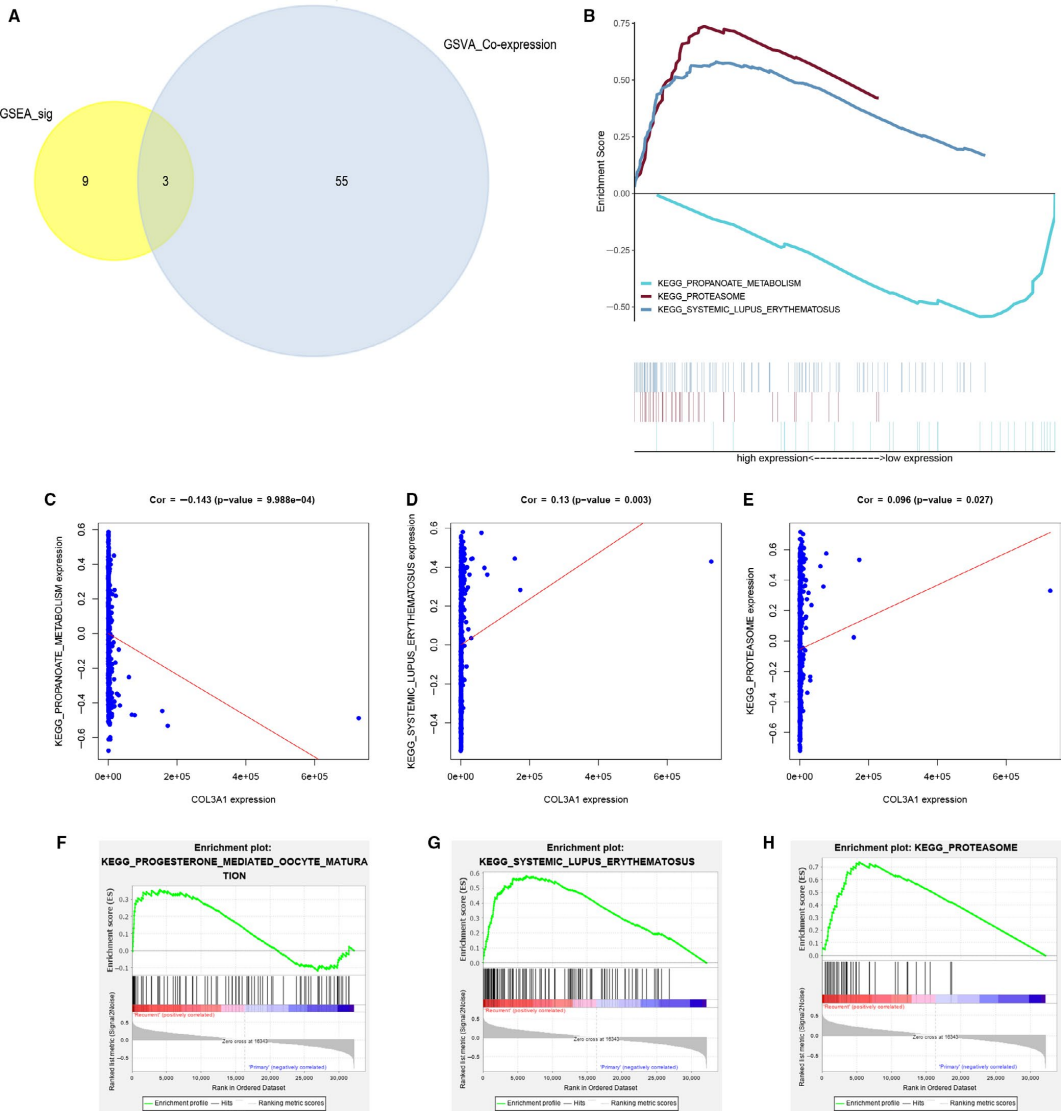
Venn plot for KEGG pathway selected by GSEA and GSVA analysis (A), GSEA analysis for top three KEGG pathways (B). Correlation analysis for propanoate metabolism pathway and COL3A1 (C), correlation analysis for systemic lupus erythematosus pathway and COL3A1 (D), correlation analysis for proteasome pathway and COL3A1 (E) by GSEA analysis. GSEA analysis for propanoate metabolism pathway (F), systemic lupus erythematosus pathway (G) and proteasome pathway (H). GSEA, Gene Set Enrichment Analysis; GSVA, Gene Set Variation Analysis; KEGG, Kyoto Encyclopedia of Genes and Genomes

A total of 184 KEGG pathways were screened by GSEA, and 12 KEGG pathways were significant differential expressed between primary and recurrent LGG samples. And Venn plot (Figure 6A) showed the 3 KEGG pathways significant in GSEA (12 KEGG pathways) and GSVA (58 KEGG pathways): propanoate metabolism, systemic lupus erythematosus and proteasome. And all significant KEGG pathways were analysed by GSEA (Figure 6B). Besides, for the relationship with COL3A1, propanoate metabolism (*P* < 0.001) (Figure 6C), systemic lupus erythematosus (*P* = 0.003) (Figure 6D) and proteasome (*P* = 0.027) (Figure 6E) were all significant. Moreover, plots of propanoate metabolism (Figure 6F), systemic lupus erythematosus (Figure 6G) and proteasome (Figure 6H) based on GSEA were also demonstrated. Additionally, according to co‐analysis with COL3A1 in heatmap, propanoate metabolism was selected form propanoate metabolism (*R* = −0.140, *P* < 0.001), systemic lupus erythematosus (R = 0.130, *P* < 0.001) and proteasome (*R* = 0.100, *P* < 0.001).

**FIGURE 7:**
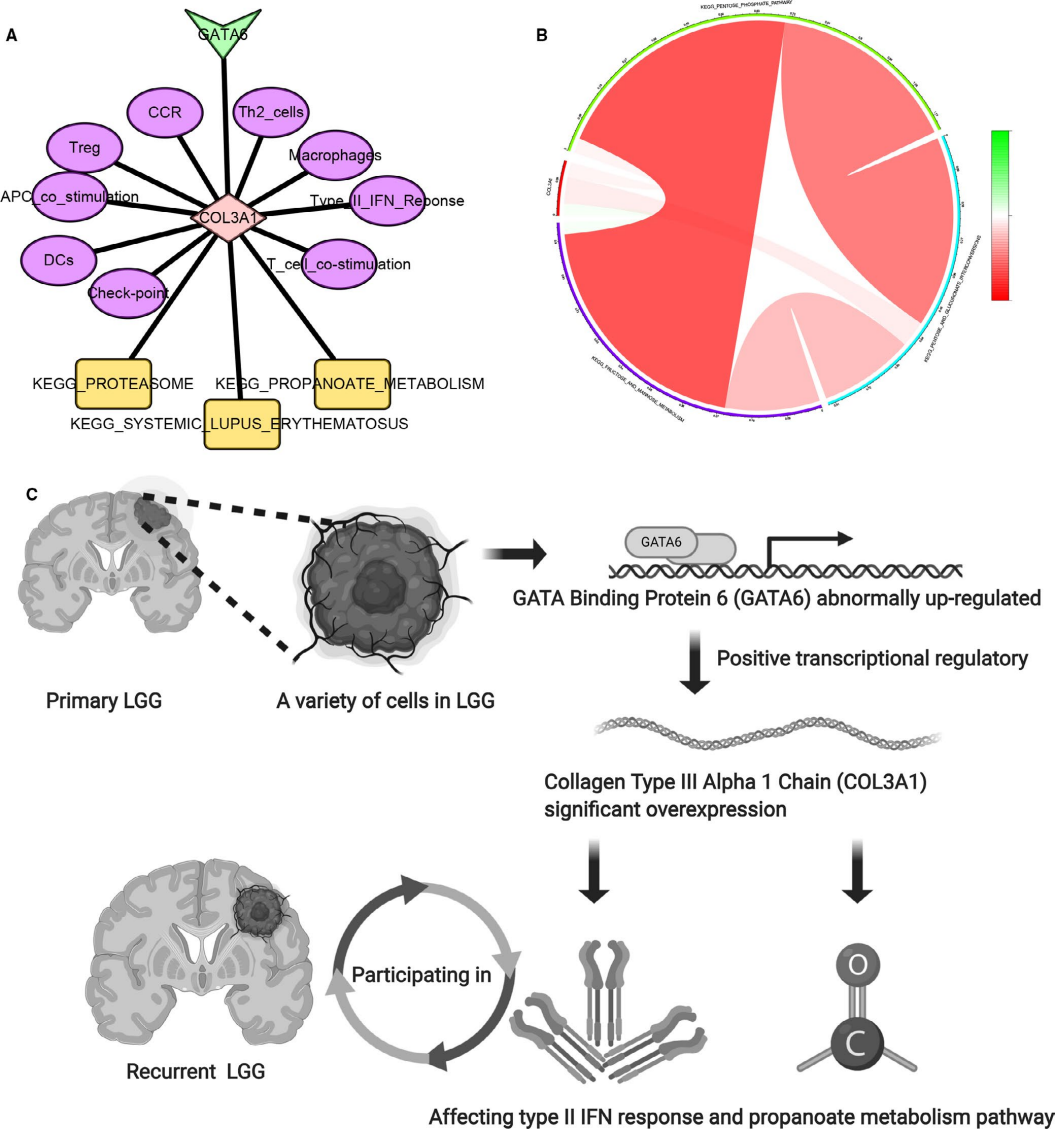
Network of TF, immune gene, immune cells/pathways and KEGG pathways (A), arrow represents TF, diamond represents immune gens, ellipses represent immune cells/pathways and rectangles represent KEGG pathways. Co‐expressed circle plot of KEGG pathways (B). Schematic diagram of all scientific hypothesis about COL3A1 Regulated by GATA6 affects type II IFN response and propanoate metabolism in the recurrence of lower grade glioma based on analysis in silico (C). KEGG, Kyoto Encyclopedia of Genes and Genomes; TF, transcription factor

Therefore, the regulatory network (Figure 7A) based on GATA6 (the most significant TF), COL3A1 (DEIG), CCR (immune pathway), Treg (immune cell), APC‐co‐stimulation (immune pathways), DC (immune cell), check‐point (immune pathway), T‐cell co‐stimulation (immune pathway), type II IFN response (immune pathway), macrophage (immune cell), Th2 cell (immune cell), propanoate metabolism (KEGG pathway), systemic lupus erythematosus (KEGG pathway) and proteasome (KEGG pathway) was established. Co‐expressed circle plot (Figure 7B) of COL3A1, propanoate metabolism, systemic lupus erythematosus and proteasome illustrated the interplay relationship.

Ultimately, the scientific hypothesis (Figure 7C): COL3A1 was positively regulated by GATA6 and affect type II IFN response and propanoate metabolism in recurrent LGG.

### 3.6 Multidimensional validation

To decrease the bias of different platform and validate our scientific hypothesis, multivariate validation was utilized. Based on immune gene set and Pathway Card, GPR146, SELP and AHR represented type II IFN response, SELP, AHR, BCKDHA, BCKDHB, DLD, SUCLG1 and DBT represented propanoate metabolism, respectively. GEPIA (Figure S2), Oncomine (Figure S3), PROGgeneV2 (Figure S4), UALCAN (Figure S5), Linkedomics (Figure S6), cBioportal (Figure S7), GTEx (Figure S8), UCSC xena (Figure S9), CCLE (Figure S10), Expression atlas, The human protein atlas (Figure S11), CGGA (Figure S12) and String (Figure S13) database were validated. Gene relationships were summarized in Table S1, all expression levels were summarized in Table S2, and all prognosis information were summarized in Table S3. All these figures and tables were mentioned in supplement materials.

GATA6 and SELP low‐expressed and AHR, BCKDHA, BCKDHB, DLD, SUCLG1 and DBT high‐expressed in LGG.

GATA6 (*P* = 0.007, Linkedomics, Figure S6A), COL3A1 (*P* < 0.001, GEPIA, Figure S2L; *P* = 0.007, PROGgeneV2, Figure S4B; *P* < 0.001, Linkedomics, Figure S6B), AHR (*P* = 0.014, Linkedomics, Figure S6E), BCKDHA (*P* = 0.011, PROGgeneV2, Figure S4E; *P* = 0.041, Linkedomics, Figure S6F; *P* = 0.013, CGGA, Figure S12F), SUCLG1 (*P* < 0.001, GEPIA, Figure S2S; *P* < 0.001, Linkedomics, Figure S6I) and DBT (*P* = 0.032, cBioportal, Figure S7T; *P* = 0.008, CGGA, Figure S12T) showed significant related to overall survival; BCKDHA (*P* = 0.021, cBioportal, Figure S7P) and DBT (*P* < 0.001, cBioportal, Figure S8R) showed significant related to prognosis; GPR146 (*P* = 0.021, CGGA, Figure S12M), SELP (*P* = 0.021, CGGA, Figure S12N) and BCKHDA (*P* = 0.012, CGGA, Figure S12P) showed significant related to recurrence.

## 4 DISCUSSION

Primary LGG develops slowly and has a well prognosis. However, the prognosis becomes poor when LGG recurs, progresses and deteriorates.^7^ And the progression‐free survival of 5‐, 10‐ and 15‐ years were 38%, 18% and 1%, respectively.^6^ Besides, the rate of malignant conversion at the first recurrence was 36.4%.^6^ Thus, a biomarker for predicting the recurrence in LGG is essential. We proposed immune genes play a role in prognosis.^22^

In our study based on comprehensive bioinformation, a total of 536 LGG samples, 2,498 immune genes and 318 TFs were acquired. Based on edgeR method, 2,164 DEGs, 2,498 DEIGs and 31 differential expressed TFs were identified. A total of 106 DEIGs from univariate Cox regression were integrated into multivariate prognostic model. Additionally, the AUC of the ROC curve was 0.860, and Kaplan‐Meier curve *P* value < 0.001. And univariate (HR = 1.007, 95% CI (1.004‐1.009), *P* < 0.001) and multivariate (HR = 1.006, 95% CI (1.003‐1.008), *P* < 0.001) Cox regression model showed risk score from multivariate prognostic model was an independent prognostic factor. Further, according to 14 clinical‐related DEIGs and significant TF‐immune gene links, GATA6 (TF) and COL3A1 (DEIG) were selected (*R* = 0.900, *P* < 0.001, positive). Moreover, 4 immune cells and 5 immune pathways were selected by ssGSEA. And based on co‐analysed COL3A1 and 29 types of immune cells or pathways, type II IFN response (*R* = 0.300, *P* < 0.001) was significant. What’ s more, based on the comprehensive consideration of GSEA, GSVA and co‐analysis result of COL3A1 and 184 KEGG pathways, propanoate metabolism (*R* = −0.140, *P* < 0.001) was selected. Eventually, COL3A1 was positively regulated by GATA6, and type II IFN response and propanoate metabolism were the downstream of COL3A1 in recurrent LGG.

GATA6 is a TF named GATA‐binding protein 6, which belongs to the GATA family, and it expressed in neuron, astrocyte, choroid plexus epithelial cells and endothelial cells.^23^ GATA6 plays an important role in differentiation during the process of embryogenesis, in proliferation during the process of development, and in inhibition apoptosis during the process tumour genesis.^24^ In addition, the frequency of methylation in GATA6 was 68.4%, and hypermethylation in GATA6 was positively related to the prognosis of glioblastoma multiforme (GBM).^25^ Besides, based on expression, loss of heterozygosity and loss function analysis, GATA6 low‐expressed in tumour, and researcher indicated that GATA6 was a new tumour suppressor gene in astroglioma.^26^ However, GATA6 also plays a different role in promoting recurrence in cholangiocarcinoma and related to poor prognosis.^27^

COL3A1 is an immune gene named collagen type III alpha 1 chain, and it codes the pro‐alpha 1 chains of type III collagen in the vascular system and other tissue.^28^ COL3A1 highly expressed in glioma.^1^ Some study indicated that it promoted tumour genesis by regulating the angiogenesis process, and it was defined as an oncogene.^28^, ^29^ By validation based on qPCR, COL3A1 correlated with prognosis of GBM, and the expression level was related to the grade of glioma (normal vs LGG, *P* < 0.001; normal vs higher grade glioma (HGG), *P* = 0.002; LGG vs HGG, *P* = 0.005).^1^ And some studies showed it might relate to recurrence in LGG by genome‐scale integrated analysis.^29^

However, there was no study reported the direct interaction between GATA6 and COL3A1. Based on bioinformation analysis and other studies,^27^, ^29^ we speculated GATA6 played a role in eliciting the transcription of COL3A1, and high‐expressed COL3A1 coding more pro‐alpha 1 chains of type III collagen in vessel, then activated angiogenesis process which participate in recurrence of LGG.

Type II IFN response is a kind of defence mechanism, and the main molecule was IFN‐γ. However, IFN‐γ can positively and negatively regulate immune system; thus, it is an antitumour cytokine and also a promoter for tumour genesis.^30^ And in centre nervous system, IFN‐γ mediated neurogenesis and differentiation through ERK1/ 2, and it also plays a role in apoptosis in brain cells and blastoma.^31^ Besides, it promoted PD‐L1 expression and inhibited the immune system, which inhibited tumour eradication by T cells and promoted immune escape.^32^

IFN‐γ can inhibit tumour angiogenesis to inhibit tumour genesis, and ICAM1 low‐expressed when knockout IFN‐γ.^33^ By String database^21^ analysis, COL3A1 regulates biomarkers of type II IFN response (GPR146, SELP and AHR) by ICAM1 and VCAM1. No study has shown the relationship between COL3A1 and IFN‐γ, but we assume COL3A1 down‐regulated type II IFN response to promote angiogenesis in tumour.

Propanoate metabolism is downstream of the lipid metabolism, and propanoate will convert into succinyl‐coenzyme A; then, the latter will enter the tricarboxylic acid cycle to supply energy or enhance lipogenesis. Based on Pathway Card (https://pathcards.genecards.org/ ), the main genes in propanoate metabolism are BCKDHA, BCKDHB, DLD, SUCLG1 and DBT. And the propanoate metabolism can be a carbon source for fatty acid biosynthesis and promote cell multiplication in pancreatic carcinoma.^34^

In tumour genesis, propanoate metabolism plays a role in lipogenesis, and by down‐regulating propanoate metabolism and lipogenesis, leading to inhibition of angiogenesis in vitro/in vivo.^35^ However, COL3A1 elicits the process of angiogenesis.^28^ Besides, ICAM1 is induced by IFN‐γ, and IFN‐γ inhibits angiogenesis.^33^ And String database^21^ analysis, COL3A1 regulates biomarkers of propanoate metabolism (BCKDHA, BCKDHB, DLD, SUCLG1 and DBT) by ICAM1 and VCAM1. Although no study indicated COL3A1 regulated propanoate metabolism, we speculated COL3A1 down‐regulated propanoate metabolism to promote lipogenesis, thus promoting angiogenesis in tumour genesis and recurrence.

All in all, in our hypothesis, GATA6 was the most significant TF and COL3A1 was the most significant DEIG. And COL3A1 was positively regulated by GATA6, and have an influence on recurrence of LGG through two potential downstream pathways: type II IFN response and propanoate metabolism. However, the inherent vice of analysis in silico was bias base on different platform, and our scientific hypothesis only based on bioinformation instead of mechanism exploring.

Therefore, multidimensional validation was applied to validate our hypothesis in different databases. Additionally, a serial experiment according to some studies to validate our hypothesis will be launched.^36^ Firstly, expression level of GATA6, COL3A1 and biomarker of type II IFN response and propanoate metabolism will be detected by immunohistochemistry (IHC) in normal brain tissue, adjacent tissue and LGG tissue. Secondly, gain/loss of function experiment was utilized to explore the regulatory relationship between these genes and phenotype of recurrence. Thirdly, to explore direct interplay of COL3A1 and GATA6, COL3A1 and type II IFN response, COL3A1 and propanoate metabolism, chromatin immunoprecipitation, luciferase reporter assay, RNA immunoprecipitation and co‐immunoprecipitation will be applied. Besides, to explore the regulatory relationship, gain/loss of function experiment was utilized. Ultimately, the indirect regulatory relationship including GATA6‐COL3A1‐type II IFN response and GATA6‐COL3A1‐propanoate metabolism was validated by rescue assays. All our effort might offer new biomarkers to prognosis the recurrence in LGG.

Based on analysis in silico and multidimensional validation, we proposed that COL3A1 was positively regulated by GATA6, and by affecting type II IFN response and propanoate metabolism, COL3A1 involved in recurrence LGG. And the prognosis value of GATA6, COL3A1, type II IFN response and propanoate metabolism might play a role in recurrence mechanism.

## CONFLICT OF INTEREST

The authors declare that there is no potential conflict of interest and no commercial or financial relationships in this study.

## AUTHOR CONTRIBUTIONS

Conception/design: Runzhi Huang, Zhenyu Li, Xiaolong Zhu, Penghui Yan, Dianwen Song, Huabin Yin, Peng Hu, Ruoyi Lin, Shengyu Wu, Tong Meng, Jie Zhang, Zongqiang Huang. Collection and/or assembly of data: Runzhi Huang, Zhenyu Li, Xiaolong Zhu, Penghui Yan, Dianwen Song, Huabin Yin, Peng Hu, Ruoyi Lin, Shengyu Wu. Data analysis and interpretation: Runzhi Huang, Zhenyu Li, Xiaolong Zhu, Penghui Yan, Dianwen Song, Huabin Yin, Peng Hu, Ruoyi Lin, Shengyu Wu, Tong Meng, Jie Zhang, Zongqiang Huang. Manuscript writing: Runzhi Huang, Zhenyu Li, Xiaolong Zhu, Penghui YanTong Meng, Jie Zhang, Zongqiang Huang. Final approval of manuscript: Runzhi Huang, Zhenyu Li, Xiaolong Zhu, Penghui Yan, Dianwen Song, Huabin Yin, Peng Hu, Ruoyi Lin, Shengyu Wu, Tong Meng, Jie Zhang, Zongqiang Huang.

## ETHICAL APPROVAL

The study was approved by the Ethics Committee of First Affiliated Hospital of Zhengzhou University. The authors declare that there is no conflict of interests.

## Supporting information

Fig S1

Fig S2

Fig S3

Fig S4

Fig S5

Fig S6

Fig S7

Fig S8

Fig S9

Fig S10

Fig S11

Fig S12

Fig S13

Fig S14

Fig S15

Table S1‐S3

## Data Availability

The datasets generated and/or analysed during the current study are available in the in the Supplementary Material and TCGA‐LGG program (https://portal.gdc.cancer.gov).
