# Supplementary figures and images for: Collagen Type III Alpha 1 chain regulated by GATA‐Binding Protein 6 affects Type II IFN response and propanoate metabolism in the recurrence of lower grade glioma
Source: J Cell Mol Med. 2020 Aug 5;24(18):10803–15. doi: 10.1111/jcmm.15705 (PMC7521258; doi:10.1111/jcmm.15705)

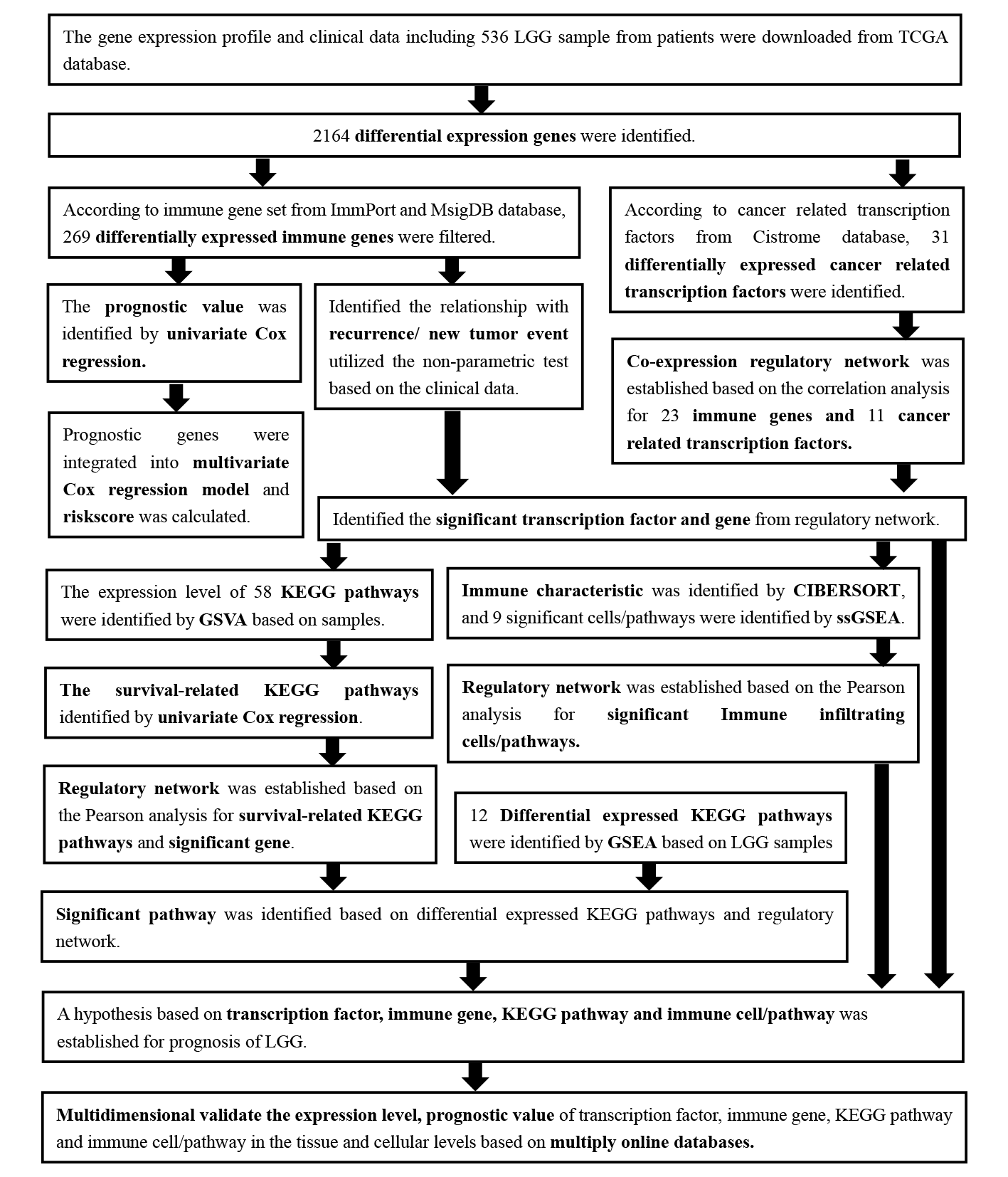

Supplement: Supplementary file 1 — Fig S1 [file JCMM-24-10803-s001.tif]

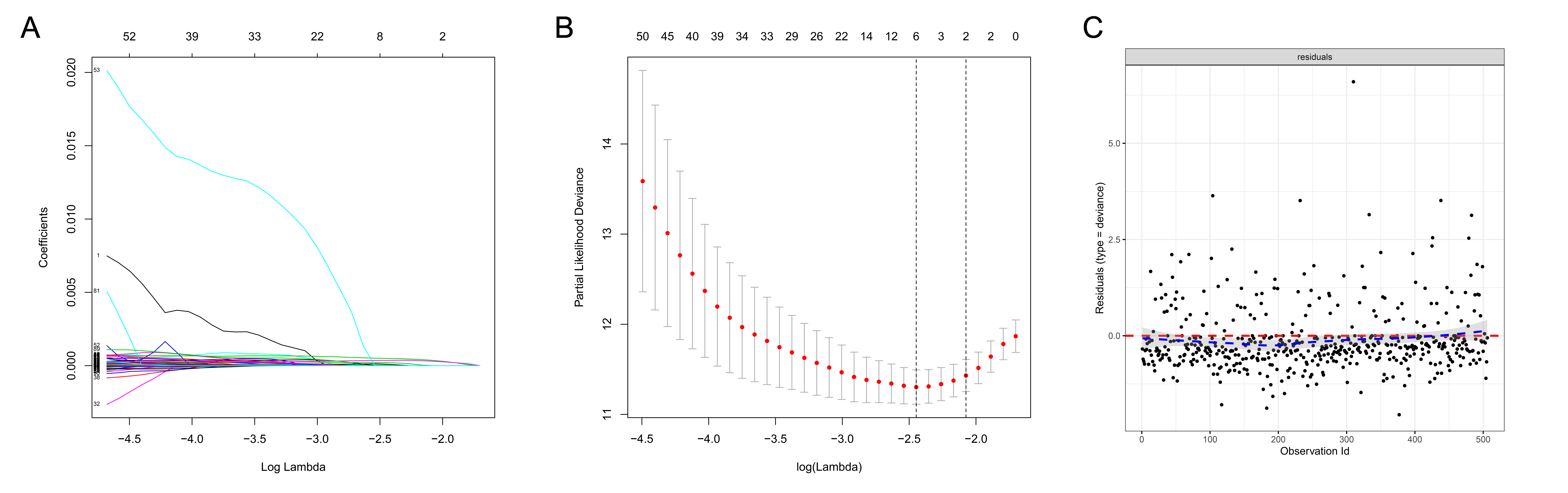

Supplement: Supplementary file 2 — Fig S2 [file JCMM-24-10803-s002.tif]

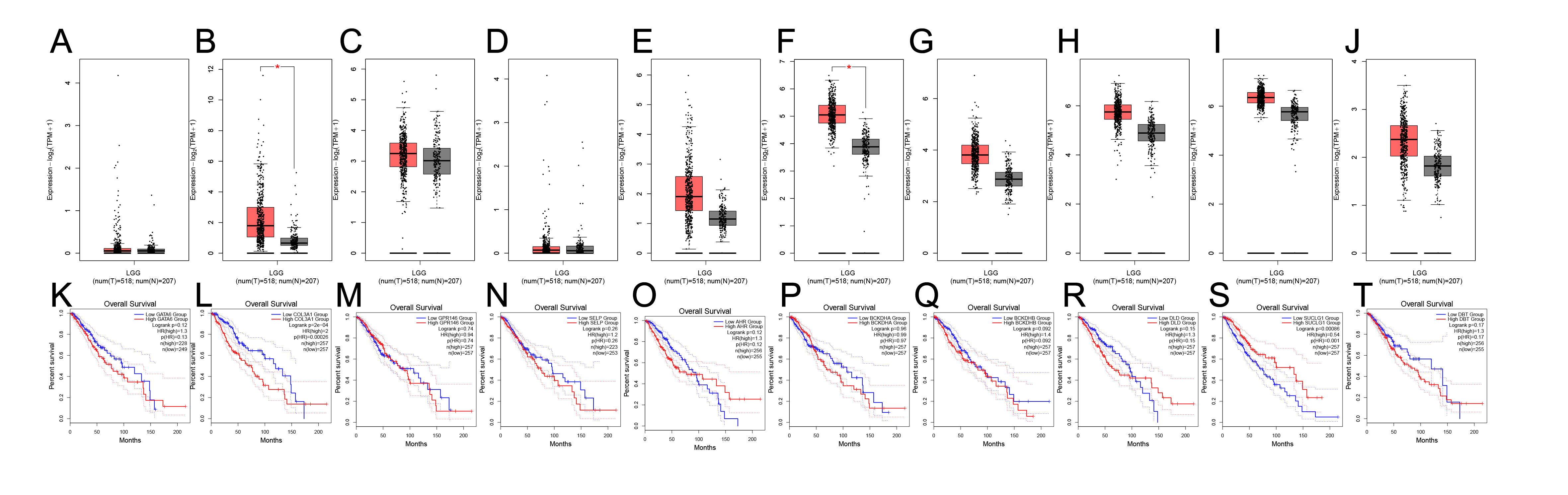

Supplement: Supplementary file 3 — Fig S3 [file JCMM-24-10803-s003.tif]

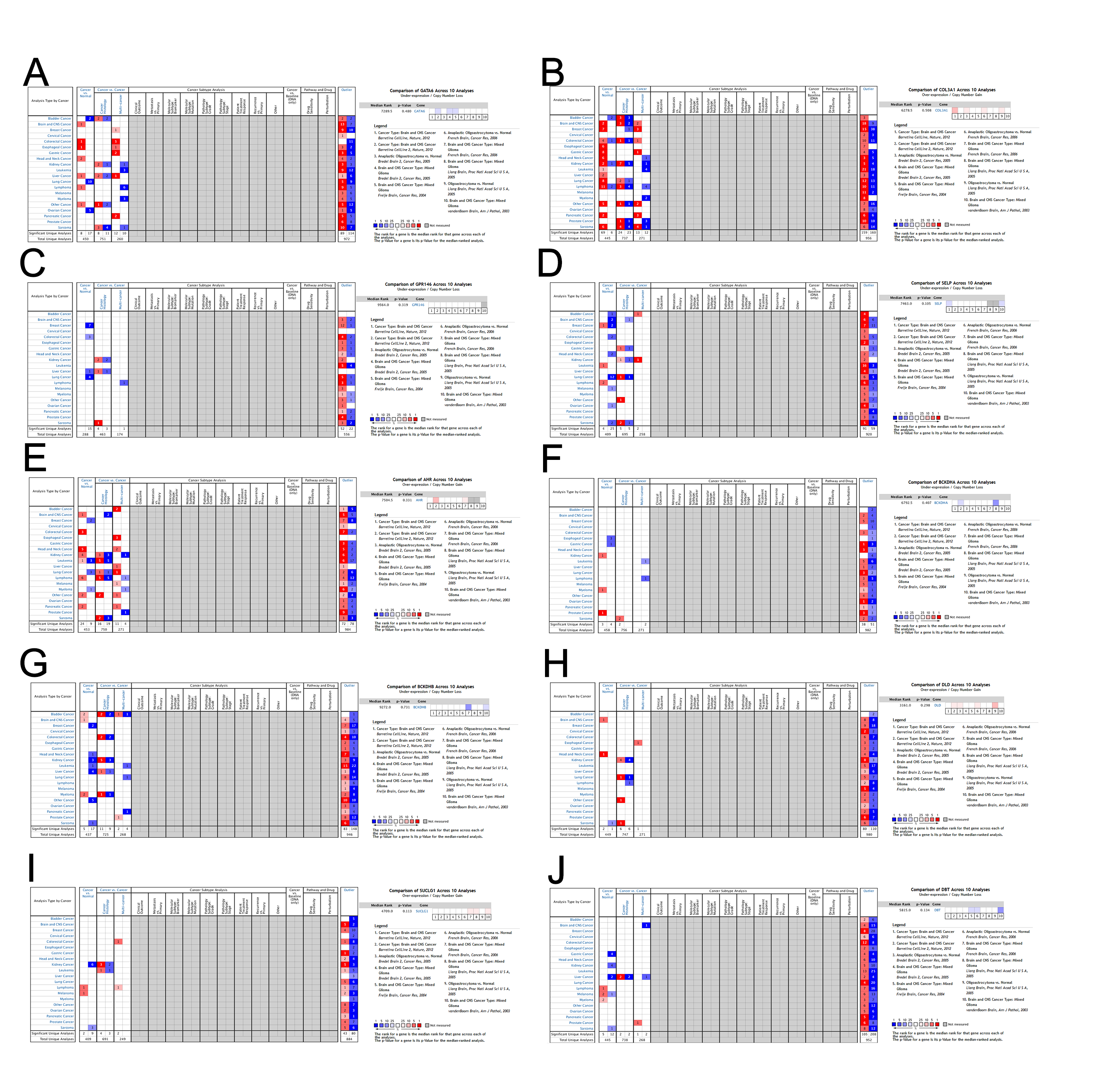

Supplement: Supplementary file 4 — Fig S4 [file JCMM-24-10803-s004.tif]

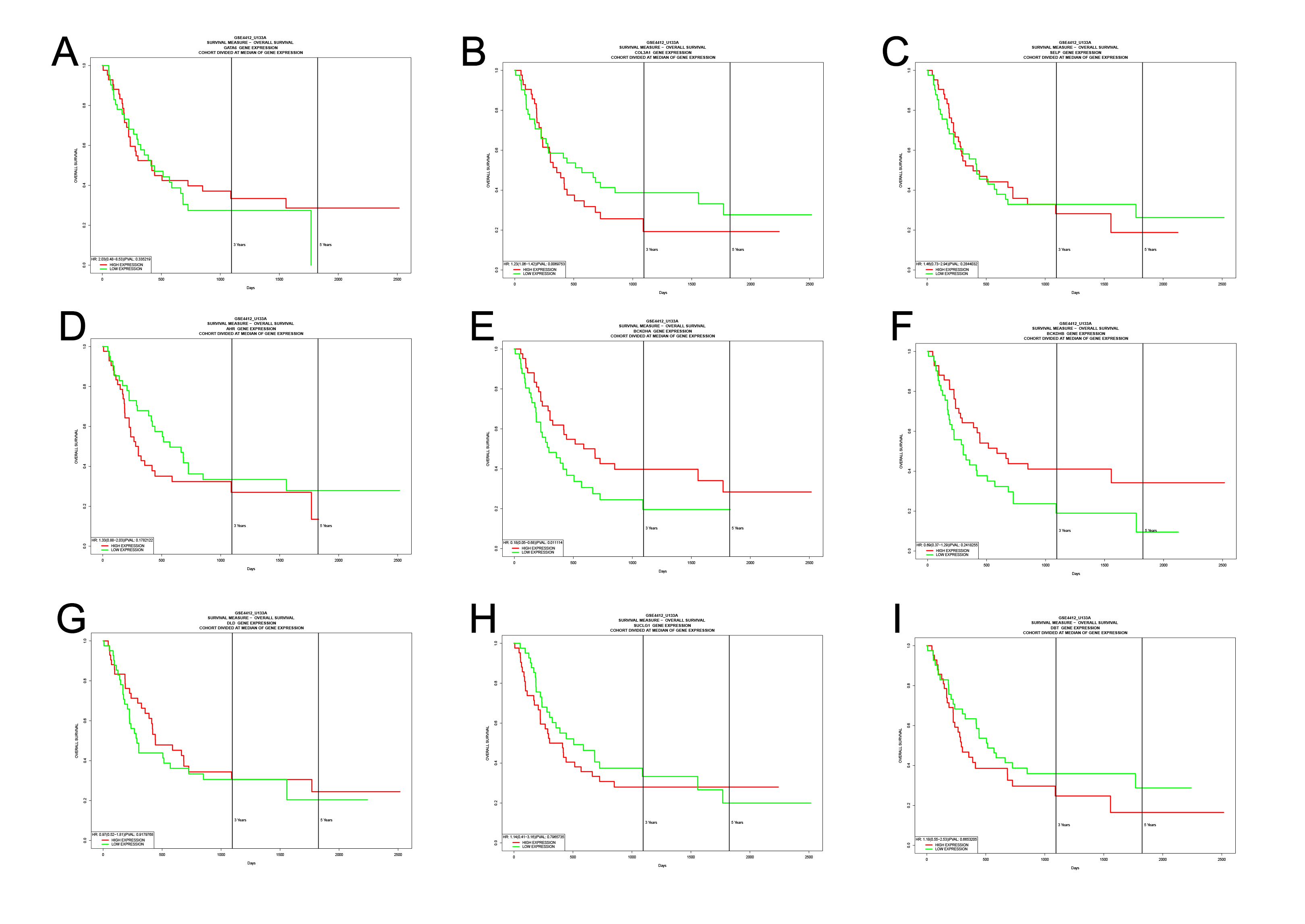

Supplement: Supplementary file 5 — Fig S5 [file JCMM-24-10803-s005.tif]

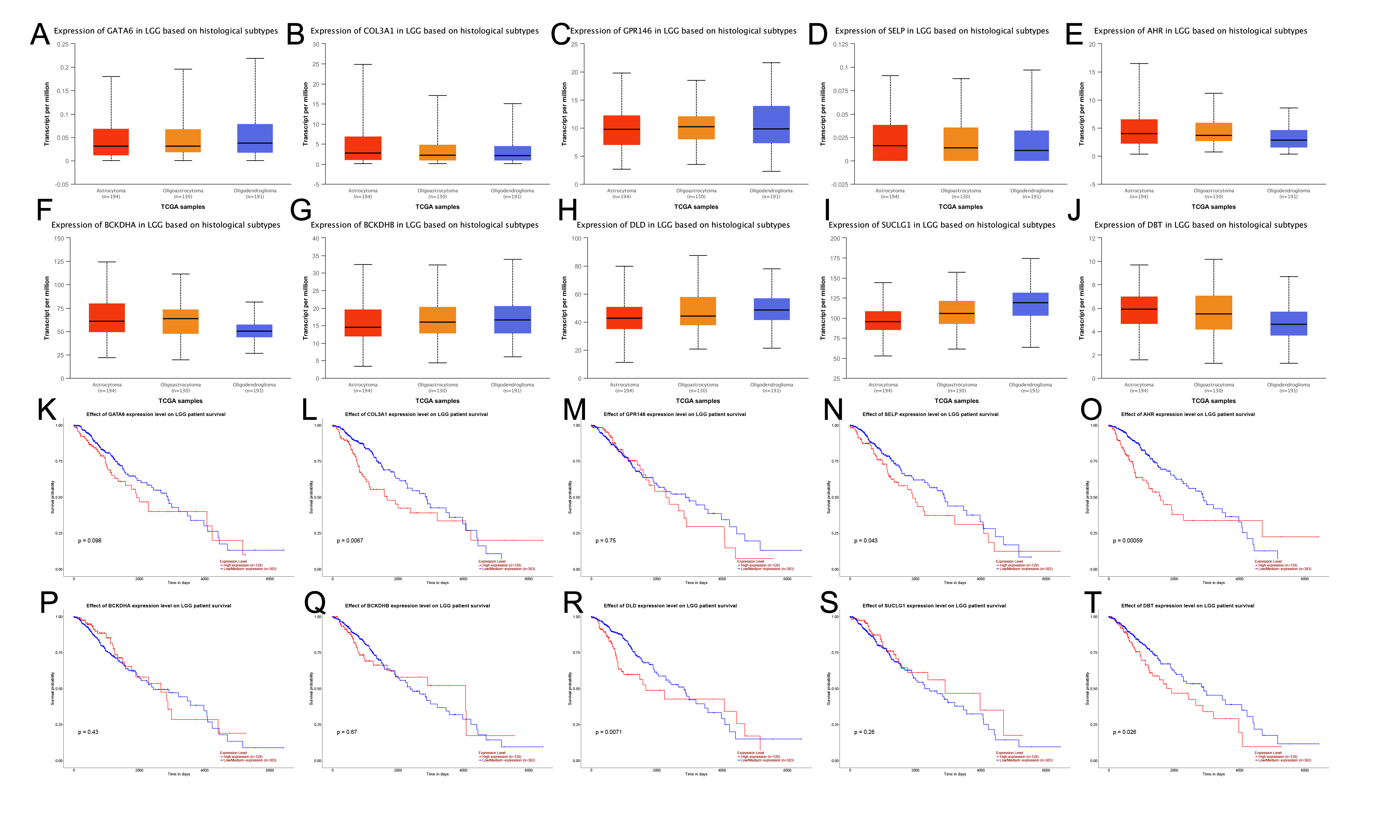

Supplement: Supplementary file 6 — Fig S6 [file JCMM-24-10803-s006.tif]

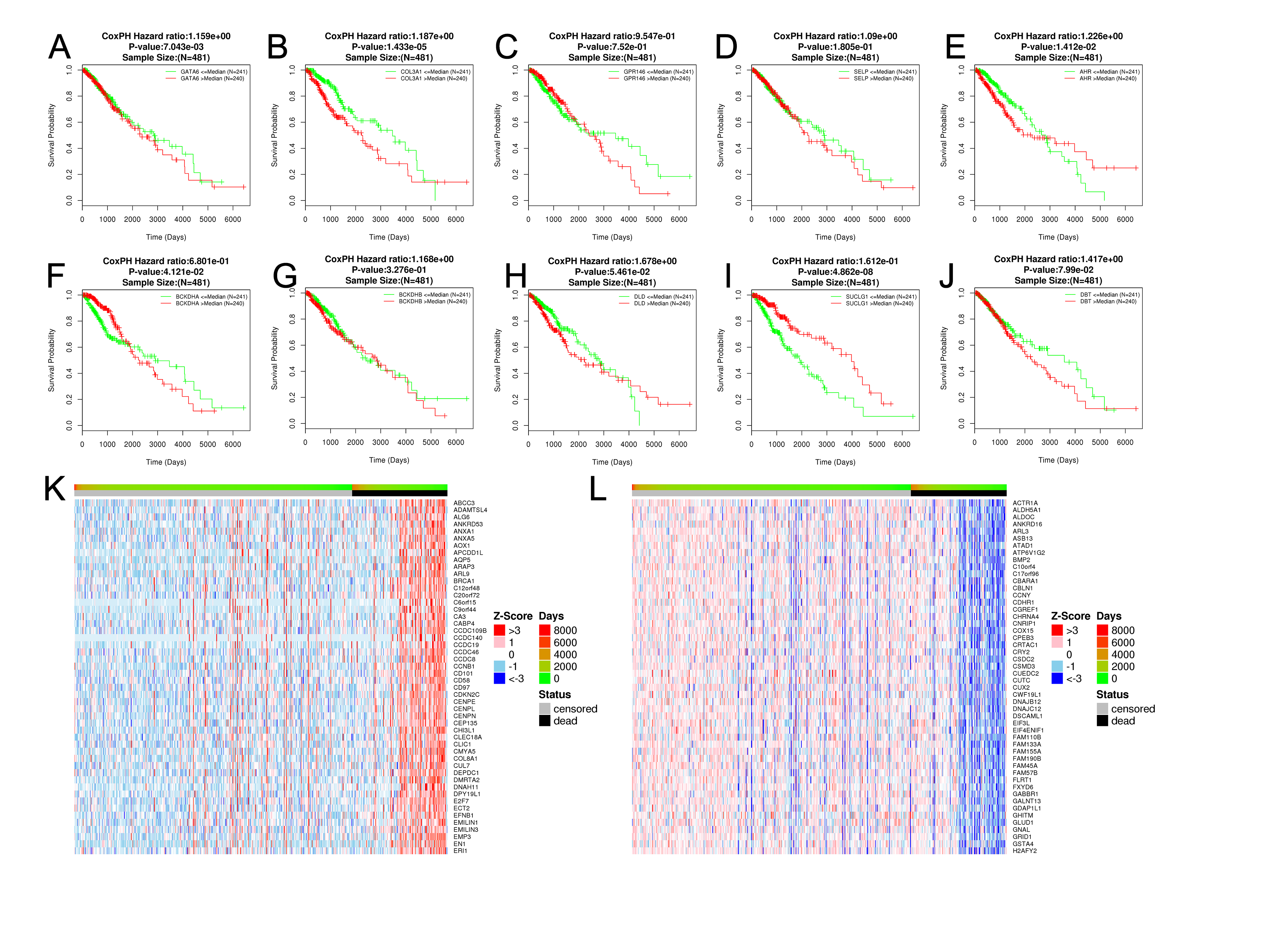

Supplement: Supplementary file 7 — Fig S7 [file JCMM-24-10803-s007.tif]

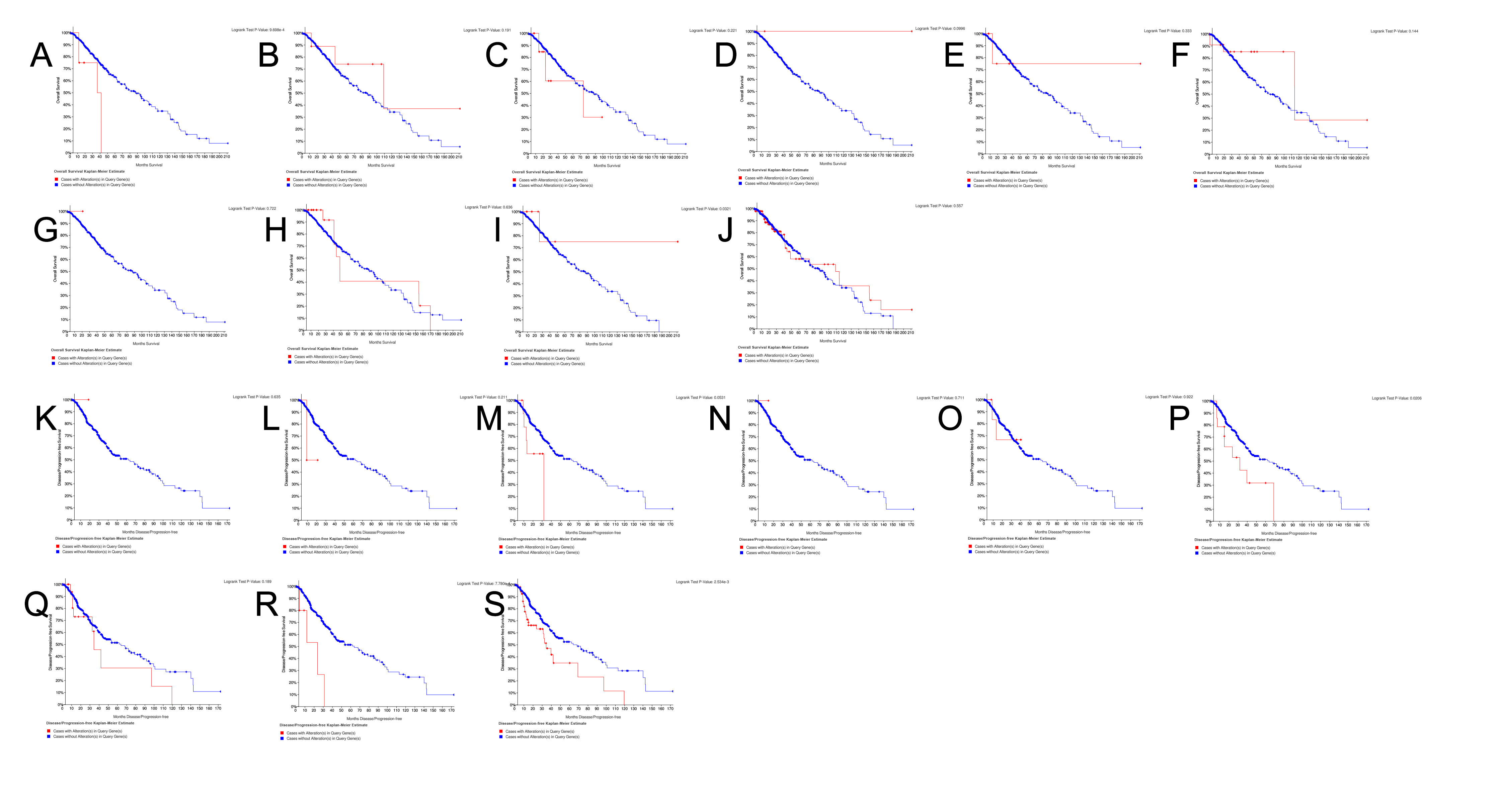

Supplement: Supplementary file 8 — Fig S8 [file JCMM-24-10803-s008.tif]

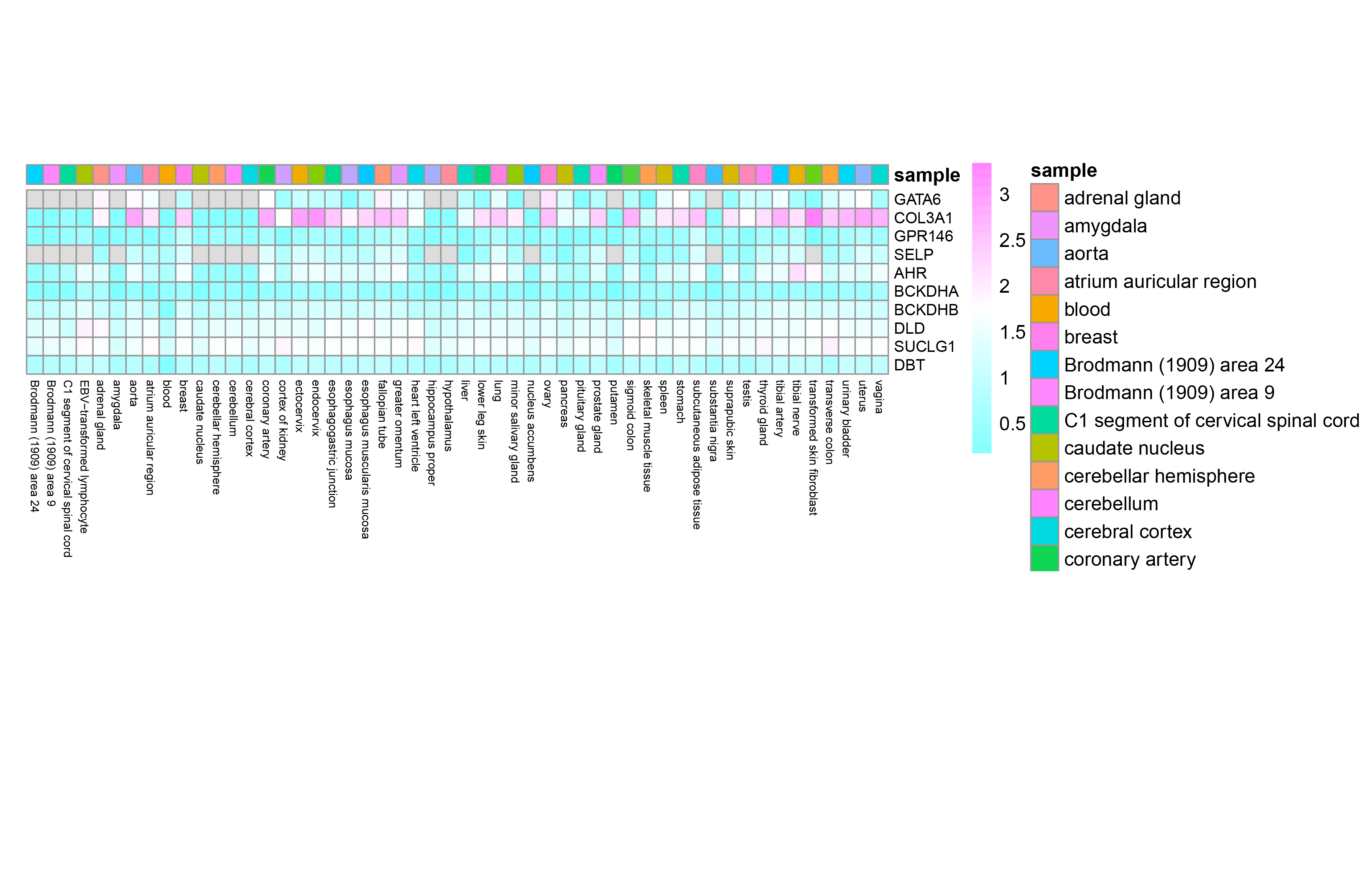

Supplement: Supplementary file 9 — Fig S9 [file JCMM-24-10803-s009.tif]

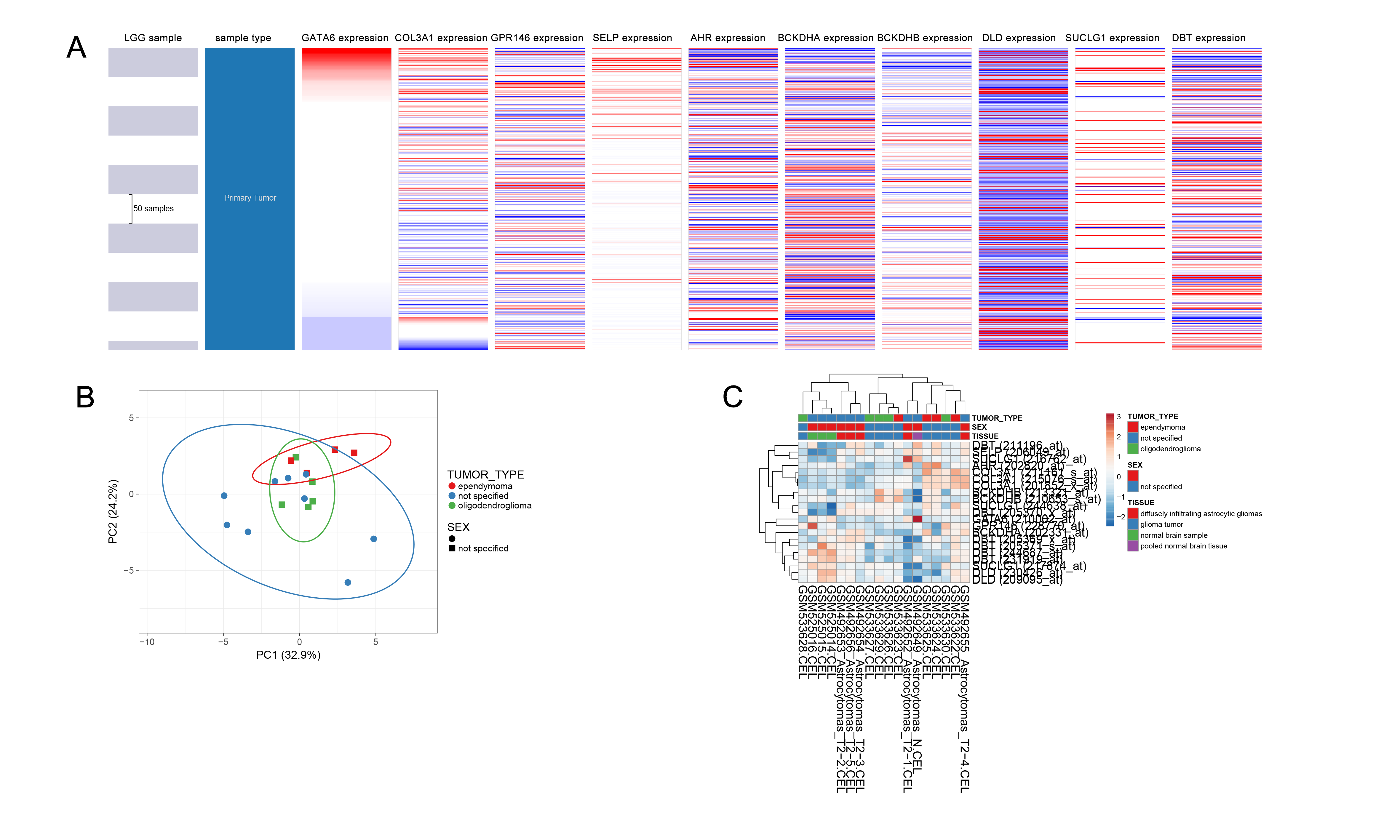

Supplement: Supplementary file 10 — Fig S10 [file JCMM-24-10803-s010.tif]

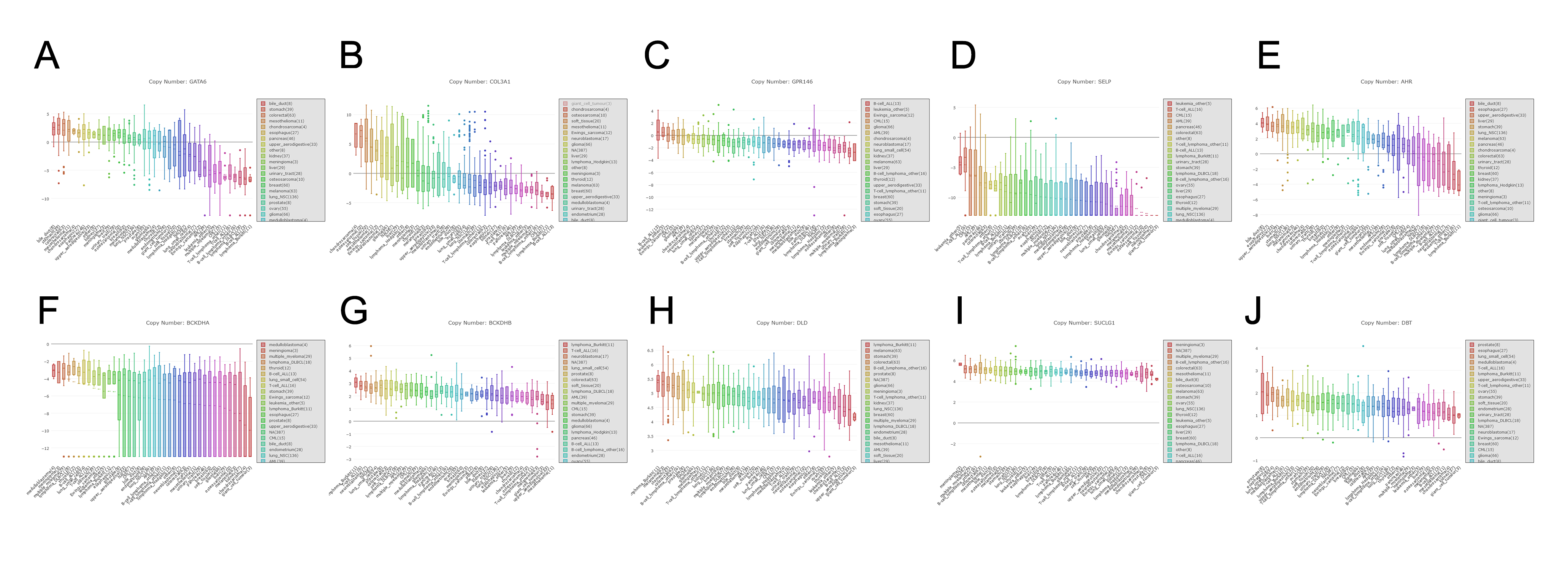

Supplement: Supplementary file 11 — Fig S11 [file JCMM-24-10803-s011.tif]

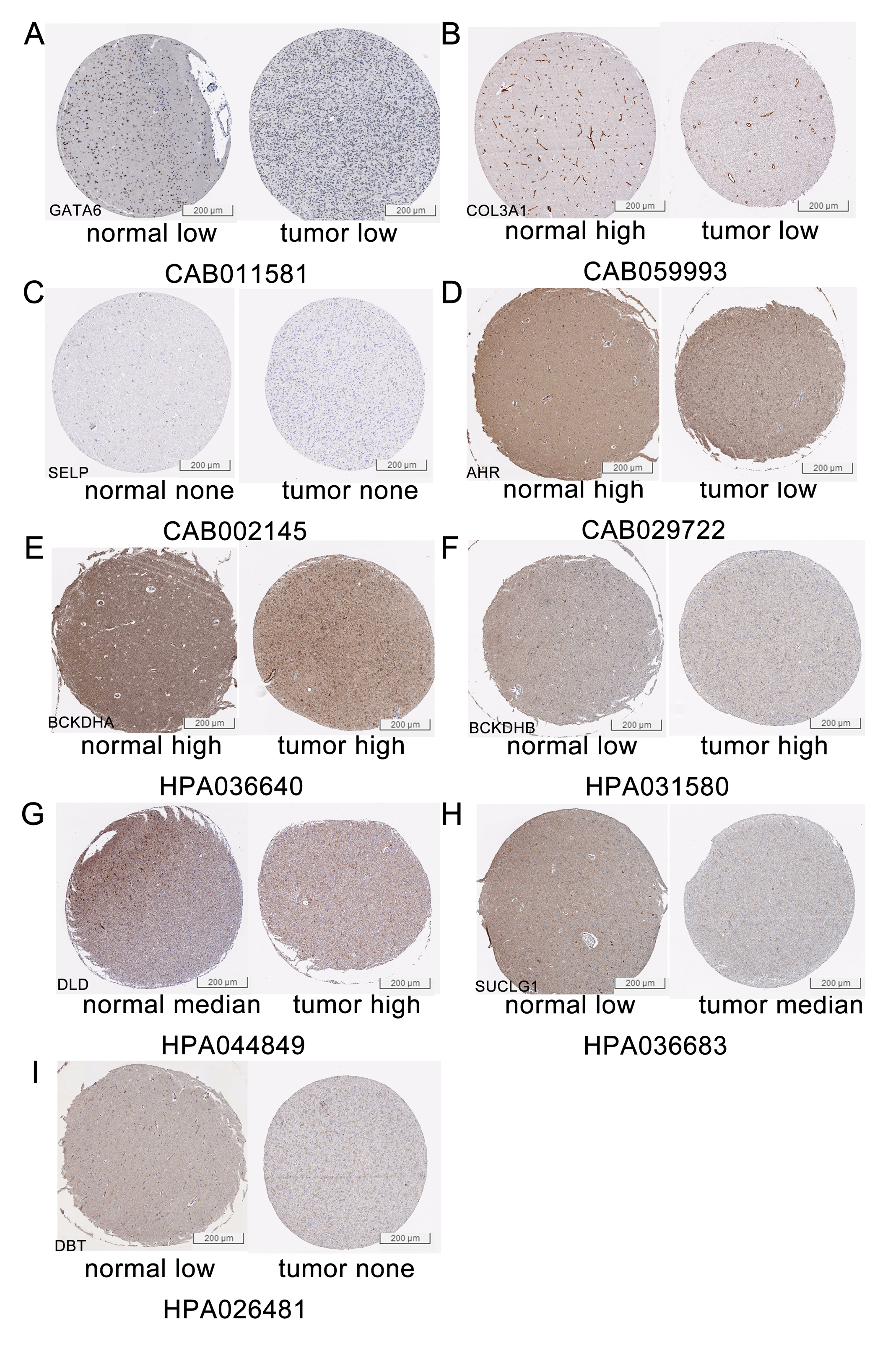

Supplement: Supplementary file 12 — Fig S12 [file JCMM-24-10803-s012.tif]

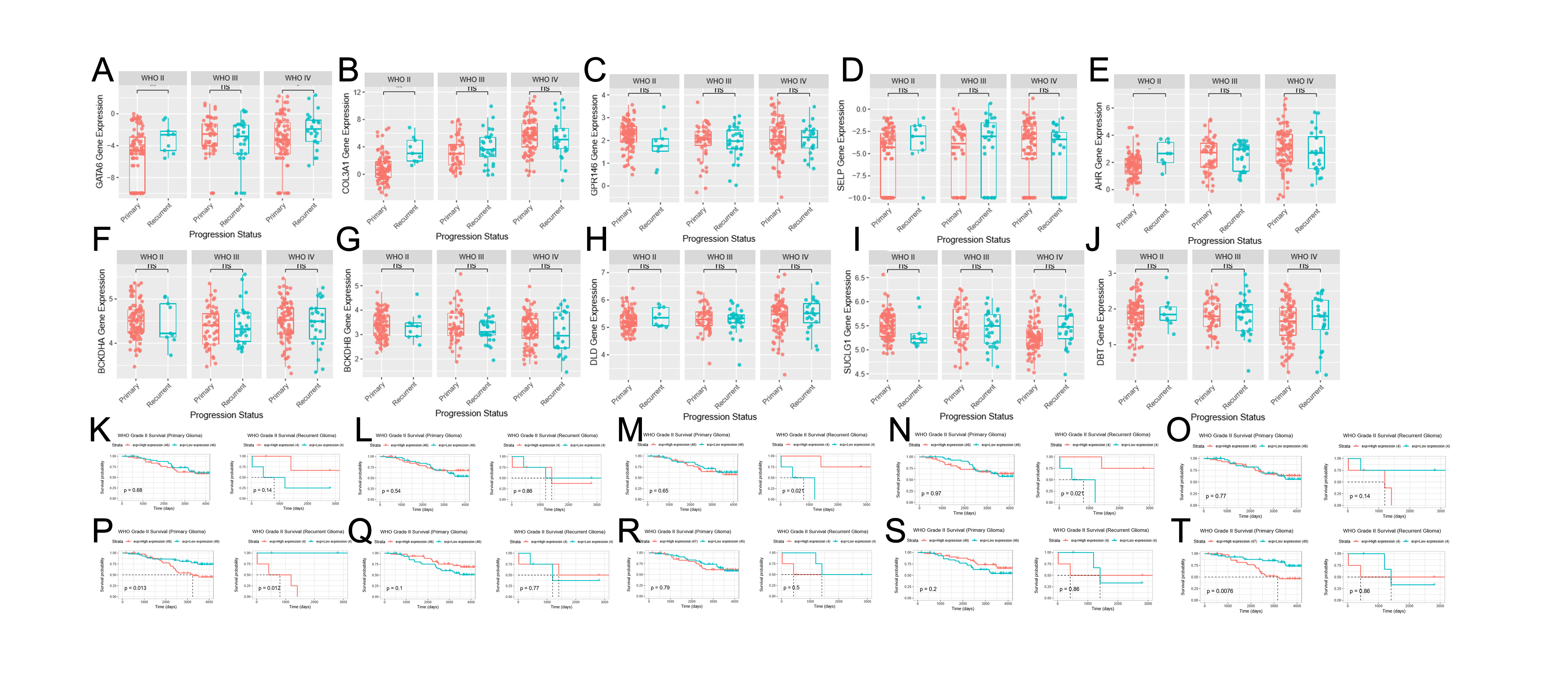

Supplement: Supplementary file 13 — Fig S13 [file JCMM-24-10803-s013.tif]

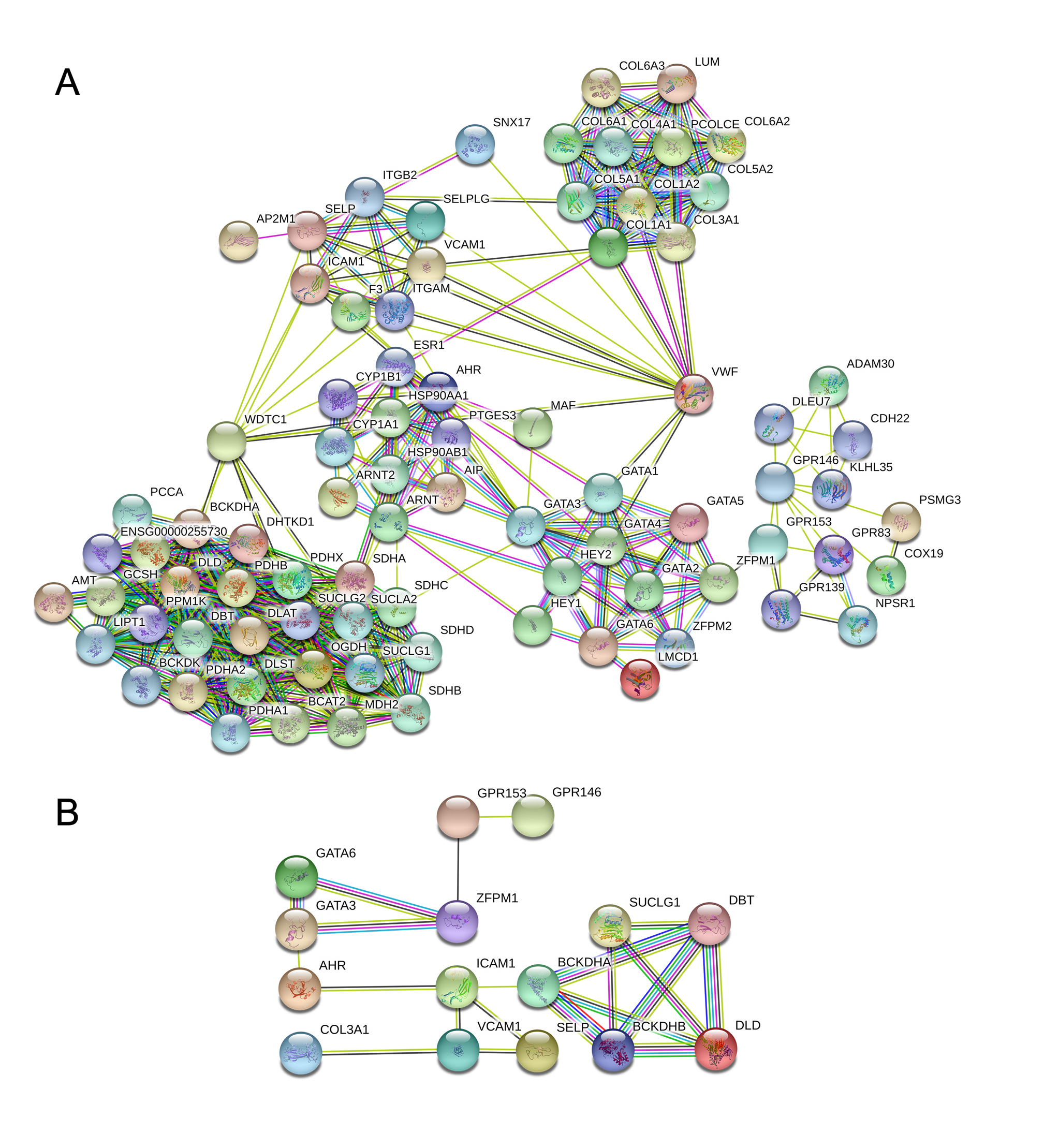

Supplement: Supplementary file 14 — Fig S14 [file JCMM-24-10803-s014.tif]

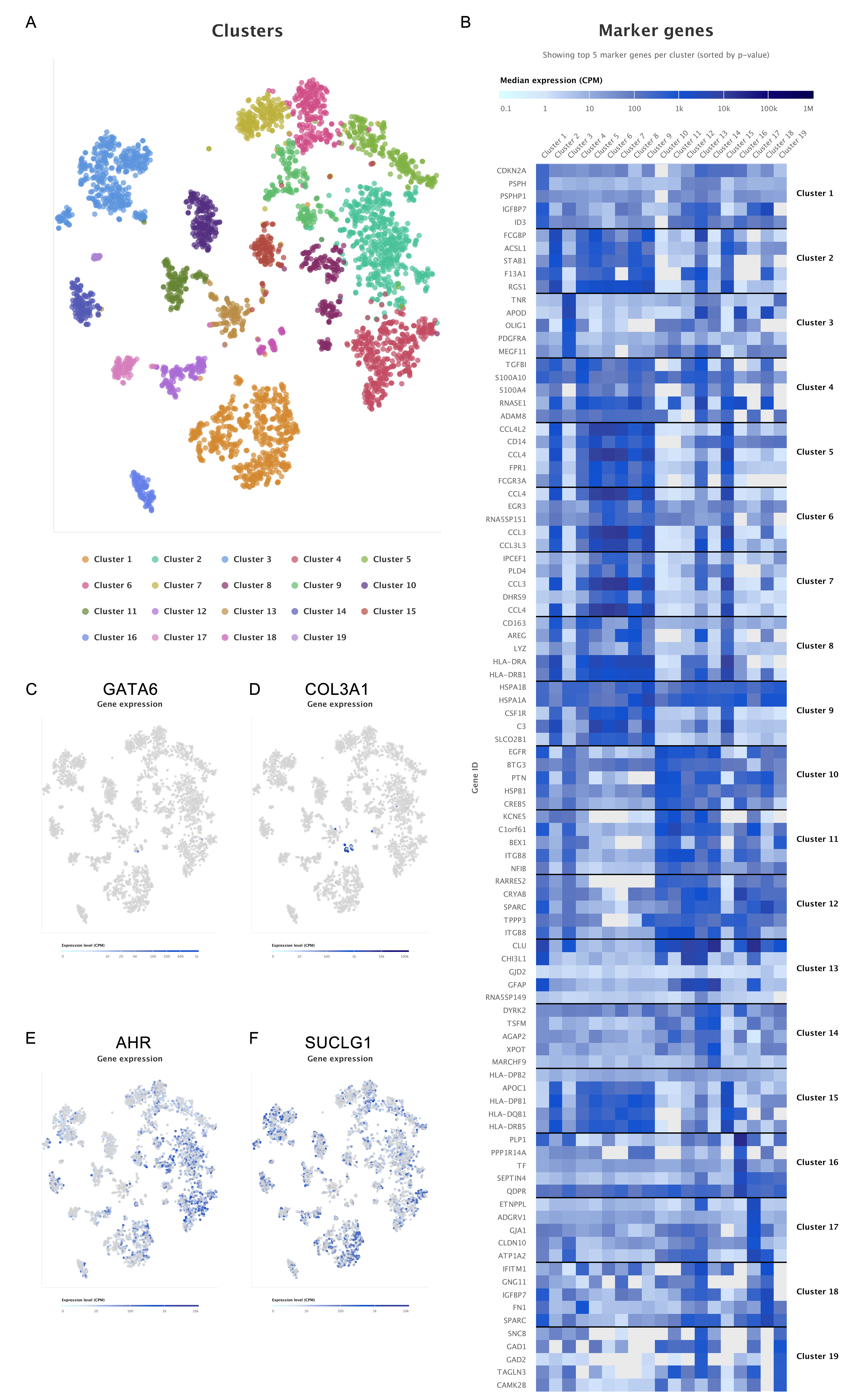

Supplement: Supplementary file 15 — Fig S15 [file JCMM-24-10803-s015.tif]
