# Supplementary material for: Collagen Type III Alpha 1 chain regulated by GATA‐Binding Protein 6 affects Type II IFN response and propanoate metabolism in the recurrence of lower grade glioma
Source: J Cell Mol Med. 2020 Aug 5;24(18):10803–15. doi: 10.1111/jcmm.15705 (PMC7521258; doi:10.1111/jcmm.15705)
Supplement: Supplementary file 16 — Table S1‐S3 [file JCMM-24-10803-s016.docx]

**Table S1.** Mutual Exclusivity of GATA6, COL3A1, GPR146, SELP, AHR, BCKDHA, BCKDHB, DLD, SUCLG1 and DBT in cBioportal database.

| **A** | **B** | **Neither** | **A Not B** | **B Not A** | **Both** | **Log2 Odds Ratio** | ***P* -Value** | **q-Value** | **Tendency** |
| --- | --- | --- | --- | --- | --- | --- | --- | --- | --- |
| BCKDHA | DBT | 828 | 18 | 5 | 2 | >3 | 0.01 | 0.353 | Co-occurrence |
| SELP | DBT | 844 | 2 | 6 | 1 | >3 | 0.024 | 0.353 | Co-occurrence |
| COL3A1 | SELP | 843 | 7 | 2 | 1 | >3 | 0.028 | 0.353 | Co-occurrence |
| SELP | AHR | 842 | 2 | 8 | 1 | >3 | 0.031 | 0.353 | Co-occurrence |
| COL3A1 | DBT | 839 | 7 | 6 | 1 | >3 | 0.064 | 0.459 | Co-occurrence |
| SELP | BCKDHA | 831 | 2 | 19 | 1 | >3 | 0.069 | 0.459 | Co-occurrence |
| AHR | DBT | 838 | 8 | 6 | 1 | >3 | 0.072 | 0.459 | Co-occurrence |
| COL3A1 | AHR | 837 | 7 | 8 | 1 | >3 | 0.082 | 0.459 | Co-occurrence |
| DLD | DBT | 834 | 12 | 6 | 1 | >3 | 0.102 | 0.475 | Co-occurrence |
| GPR146 | AHR | 834 | 10 | 8 | 1 | >3 | 0.111 | 0.475 | Co-occurrence |
| COL3A1 | DLD | 833 | 7 | 12 | 1 | >3 | 0.116 | 0.475 | Co-occurrence |
| COL3A1 | BCKDHA | 826 | 7 | 19 | 1 | 2.635 | 0.174 | 0.651 | Co-occurrence |
| AHR | BCKDHA | 825 | 8 | 19 | 1 | 2.44 | 0.193 | 0.668 | Co-occurrence |
| BCKDHA | DLD | 820 | 20 | 13 | 0 | <-3 | 0.733 | 1 | Mutual exclusivity |
| GPR146 | BCKDHA | 822 | 11 | 20 | 0 | <-3 | 0.769 | 1 | Mutual exclusivity |
| GATA6 | BCKDHA | 823 | 10 | 20 | 0 | <-3 | 0.788 | 1 | Mutual exclusivity |
| GPR146 | DLD | 829 | 11 | 13 | 0 | <-3 | 0.844 | 1 | Mutual exclusivity |
| GATA6 | DLD | 830 | 10 | 13 | 0 | <-3 | 0.857 | 1 | Mutual exclusivity |
| AHR | DLD | 831 | 9 | 13 | 0 | <-3 | 0.87 | 1 | Mutual exclusivity |
| GATA6 | GPR146 | 832 | 10 | 11 | 0 | <-3 | 0.878 | 1 | Mutual exclusivity |
| GATA6 | AHR | 834 | 10 | 9 | 0 | <-3 | 0.899 | 1 | Mutual exclusivity |
| COL3A1 | GPR146 | 834 | 8 | 11 | 0 | <-3 | 0.901 | 1 | Mutual exclusivity |
| GATA6 | COL3A1 | 835 | 10 | 8 | 0 | <-3 | 0.91 | 1 | Mutual exclusivity |
| GPR146 | DBT | 835 | 11 | 7 | 0 | <-3 | 0.913 | 1 | Mutual exclusivity |
| GATA6 | DBT | 836 | 10 | 7 | 0 | <-3 | 0.92 | 1 | Mutual exclusivity |
| SELP | DLD | 837 | 3 | 13 | 0 | <-3 | 0.955 | 1 | Mutual exclusivity |
| GPR146 | SELP | 839 | 11 | 3 | 0 | <-3 | 0.962 | 1 | Mutual exclusivity |
| GATA6 | SELP | 840 | 10 | 3 | 0 | <-3 | 0.965 | 1 | Mutual exclusivity |
| BCKDHA | BCKDHB | 832 | 20 | 1 | 0 | <-3 | 0.977 | 1 | Mutual exclusivity |
| BCKDHB | DLD | 839 | 1 | 13 | 0 | <-3 | 0.985 | 1 | Mutual exclusivity |
| GPR146 | BCKDHB | 841 | 11 | 1 | 0 | <-3 | 0.987 | 1 | Mutual exclusivity |
| GATA6 | BCKDHB | 842 | 10 | 1 | 0 | <-3 | 0.988 | 1 | Mutual exclusivity |
| AHR | BCKDHB | 843 | 9 | 1 | 0 | <-3 | 0.989 | 1 | Mutual exclusivity |
| COL3A1 | BCKDHB | 844 | 8 | 1 | 0 | <-3 | 0.991 | 1 | Mutual exclusivity |
| BCKDHB | DBT | 845 | 1 | 7 | 0 | <-3 | 0.992 | 1 | Mutual exclusivity |
| SELP | BCKDHB | 849 | 3 | 1 | 0 | <-3 | 0.996 | 1 | Mutual exclusivity |
| GATA6 | SUCLG1 | 843 | 10 | 0 | 0 | >3 | 1 | 1 | Co-occurrence |
| COL3A1 | SUCLG1 | 845 | 8 | 0 | 0 | >3 | 1 | 1 | Co-occurrence |
| GPR146 | SUCLG1 | 842 | 11 | 0 | 0 | >3 | 1 | 1 | Co-occurrence |
| SELP | SUCLG1 | 850 | 3 | 0 | 0 | >3 | 1 | 1 | Co-occurrence |
| AHR | SUCLG1 | 844 | 9 | 0 | 0 | >3 | 1 | 1 | Co-occurrence |
| BCKDHA | SUCLG1 | 833 | 20 | 0 | 0 | >3 | 1 | 1 | Co-occurrence |
| BCKDHB | SUCLG1 | 852 | 1 | 0 | 0 | >3 | 1 | 1 | Co-occurrence |
| DLD | SUCLG1 | 840 | 13 | 0 | 0 | >3 | 1 | 1 | Co-occurrence |
| SUCLG1 | DBT | 846 | 0 | 7 | 0 | >3 | 1 | 1 | Co-occurrence |

**Table S2** Summary of multidimensional external validation results of gene expression base on multiple databases

|  | **GATA6** | | **COL3A1** | | **GPR146** | | **SELP** | | **AHR** | | **BCKDHA** | | **BCKDHB** | | **DLD** | | **SUCLG1** | | **DBT** | | **Results** |
| --- | --- | --- | --- | --- | --- | --- | --- | --- | --- | --- | --- | --- | --- | --- | --- | --- | --- | --- | --- | --- | --- |
|  | **N** | **L** | **N** | **L** | **N** | **L** | **N** | **L** | **N** | **L** | **N** | **L** | **N** | **L** | **N** | **L** | **N** | **L** | **N** | **L** |  |
| **GEPIA** | **↓** | **↓** | **↓** | **↑** | **-** | **-** | **↓** | **↓** | **-** | **↑** | **↑** | **↑** | **↑** | **↑** | **↑** | **↑** | **↑** | **↑** | **↑** | **↑** | BCKDHA, BCKDHB, DLD, SUCLG1 and DBT high-expressed in normal tissue and LGG; GATA6 and SELP low-expressed in normal tissue and LGG; COL3A1 high-expressed in LGG while low-expressed in normal tissue (figure S2). |
| **Oncomine** | **NA** | **↓** | **NA** | **↑** | **NA** | **↓** | **NA** | **↓** | **NA** | **↑** | **NA** | **↓** | **NA** | **↓** | **NA** | **↑** | **NA** | **↑** | **NA** | **↓** | COL3A1, AHR, DLD, SUCLG1 and DBT high-expressed in glioma; GATA6, GPR146, SELP, BCKDHA and BCKDHB low-expressed in glioma (figure S3). |
| **PROGgeneV2** | **NA** | **-** | **NA** | **↑** | **NA** | **NA** | **NA** | **-** | **NA** | **-** | **NA** | **↑** | **NA** | **-** | **NA** | **-** | **NA** | **-** | **NA** | **-** | COL3A1 and BCKDHA high-expressed in cancer in brain tissue (figure S4). |
| **UALCAN** | **NA** | **↓** | **NA** | **↓** | **NA** | **↑** | **NA** | **↓** | **NA** | **↓** | **NA** | **↑** | **NA** | **↑** | **NA** | **↑** | **NA** | **↑** | **NA** | **↑** | GATA6, COL3A1, SELP and AHR low-expressed in LGG; GPR146, BCKDHA, BCKDHB, DLD, SUCLG1 and DBT high-expressed in LGG (figure S5). |
| **Linkedomics** | **NA** | **↓** | **NA** | **↑** | **NA** | **-** | **NA** | **-** | **NA** | **-** | **NA** | **-** | **NA** | **-** | **NA** | **-** | **NA** | **↑** | **NA** | - | COL3A1 and SUCLG1 high-expressed in LGG; GATA6 low-expressed in LGG (figure S6). |
| **cBioportal** | **NA** | **↓** | **NA** | **↑** | **NA** | **↑** | **NA** | **↓** | **NA** | **↑** | **NA** | **↑** | **NA** | **↑** | **NA** | **↑** | **NA** | **↑** | **NA** | **↑** | COL3A1, GPR146, AHR, BCKDHA, BCKDHB, DLD, SUCLG1 and DBT high-expressed in LGG; GATA6 and SELP low-expressed in LGG (figure S7). |
| **GTEx** | **ND** | **NA** | **↓** | **NA** | **↓** | **NA** | **ND** | **NA** | **↓** | **NA** | **↓** | **NA** | **↓** | **NA** | - | **NA** | **-** | **NA** | **↓** | **NA** | COL3A1, GPR146, AHR, BCKDHA, BCKDHB and DBT low-expressed in normal cerebral cortex tissue (figure S8). |
| **UCSC xena** | **NA** | **↓** | **NA** | **↑** | **NA** | **↑** | **NA** | **↓** | **NA** | **↑** | **NA** | **↑** | **NA** | **↑** | **NA** | **↑** | **NA** | **↑** | **NA** | **↑** | COL3A1, GPR146, AHR, BCKDHA, BCKDHB, DLD, SUCLG1 and DBT high-expressed in LGG; GATA6 and SELP low-expressed in LGG (figure S9). |
| **CCLE** | **NA** | **↓** | **NA** | **↑** | **NA** | **↓** | **NA** | **↓** | **NA** | **↑** | **NA** | **↓** | **NA** | **↑** | **NA** | **↑** | **NA** | **↑** | **NA** | **↑** | COL3A1, AHR, BCKDHB, DLD, SUCLG1 and DBT high-expressed in glioma cell line; GATA6, GPR146, SELP and BCKDHA low-expressed in glioma cell line (figure S10). |
| **Expression atlas** | **NA** | **↓** | **NA** | **↑** | **NA** | **↑** | **NA** | **↑** | **NA** | **↑** | **NA** | **NA** | **NA** | **↑** | **NA** | **NA** | **NA** | **NA** | **NA** | **↑** | COL3A1, GPR146, SELP, AHR, BCKDHB and DBT high-expressed in glioma; GATA6 and BCKDHA low-expressed in glioma. |
| **The human protein atlas** | **↓** | **↓** | **↑** | **↓** | **NA** | **NA** | **ND** | **ND** | **↑** | **↓** | **↑** | **↑** | **↓** | **↑** | **-** | **↑** | **↓** | **-** | **↓** | **ND** | BCKDHA high-expressed in normal cerebral cortex tissue and tumor tissue; GATA6 low-expressed in normal cerebral cortex tissue and tumor tissue; COL3A1 and AHR high-expressed in normal cerebral cortex tissue while low-expressed in tumor tissue; BCKDHB high-expressed in tumor tissue while low-expressed in normal cerebral cortex tissue; DLD high-expressed in tumor tissue; SUCLG1 and DBT low-expressed in normal cerebral cortex tissue (figure S11). |
| **CGGA** | **NA** | **↓** | **NA** | **-** | **NA** | **-** | **NA** | **↓** | **NA** | **-** | **NA** | **↑** | **NA** | **↑** | **NA** | **↑** | **NA** | **↑** | **NA** | **-** | BCKDHA, BCKDHB, DLD and SUCLG1 high-expressed in glioma; GATA6 and SELP low-expressed in glioma (figure S12). |

Note: “N” was defined as normal; “L” was defined as lower grade glioma;“↑” was defined as a significantly high-expressed gene; “↓” was defined as a significantly low-expressed gene; “NA” was defined as “Not available”; “ND” was defined as “Not detached”; “-” was defined as a gene with no significant difference in expression.

Abbreviations: LGG, lower grade glioma; GTEx, Genotype-Tissue Expression; CCLE, Cancer Cell Line Encyclopedia; GEPIA, Gene Expression Profilling Interactive Analysis; CGGA, the Chinese Glioma Genome Atlas.

**Table S3** Summary of multidimensional external validation results of prognosis based on multiple databases

|  | **GATA6** | **COL3A1** | **GPR146** | **SELP** | **AHR** | **BCKDHA** | **BCKDHB** | **DLD** | **SUCLG1** | **DBT** | **Results** |
| --- | --- | --- | --- | --- | --- | --- | --- | --- | --- | --- | --- |
| **GEPIA** | OS  *P* = 0.120 | OS  *P* ＜ 0.001 | OS  *P* = 0.740 | OS  *P* = 0.260 | OS  *P* = 0.120 | OS  *P* = 0.960 | OS  *P* = 0.092 | OS  *P* = 0.150 | OS  *P* ＜ 0.001 | OS  *P* = 0.170 | COL3A1 and SUCLG1 show significant related to overall survival (figure S2). |
| **PROGgeneV2** | OS  *P* = 0.335 | OS  *P* = 0.007 | NA | OS  *P* = 0.284 | OS  *P* = 0.178 | OS  *P* = 0.011 | OS  *P* = 0.242 | OS  *P* = 0.918 | OS  *P* = 0.797 | OS  *P* = 0.665 | COL3A1 and BCKDHA show significant related to overall survival (figure S4). |
| **Linkedomics** | OS *P* = 0.007 | OS *P*＜ 0.001 | OS *P* = 0.752 | OS *P* = 0.181 | OS *P* = 0.014 | OS *P* = 0.041 | OS *P* = 0.328 | OS *P* = 0.055 | OS *P*＜ 0.001 | OS *P* = 0.080 | GATA6, COL3A1, AHR, BCKDHA and SUCLG1 show significant related to overall survival (figure S6). |
| **cBioportal** | OS  *P*＜ 0.001  D/PF  *P* = 0.635 | OS *P* = 0.191  D/PF  *P* = 0.211 | OS *P* = 0.221  D/PF  *P* = 0.053 | OS *P* = 0.100 D/PF  *P* = 0.711 | OS *P* = 0.333  D/PF  *P* = 0.922 | OS *P* = 0.144  D/PF  *P* = 0.021 | OS *P* = 0.722  D/PF  *P* = NA | OS *P* = 0.636  D/PF  *P* = 0.189 | NA | OS *P* = 0.032  D/PF  *P*＜ 0.001 | GATA6 and DBT show significant related to overall survival; BCKDHA and DBT show significant related to prognosis; the integrated genes show significant related to prognosis (figure S7). |
|  | OS of integrated genes *P* = 0.557  D/PF *P* = 0.003 | | | | | | | | | |  |
| **CGGA** | OS *P* = 0.680  Recurrence  *P* = 0.140 | OS *P* = 0.540  Recurrence  *P* = 0.86 | OS *P* = 0.650  Recurrence  *P* = 0.021 | OS *P* = 0.970  Recurrence  *P* = 0.021 | OS *P* = 0.770  Recurrence  *P* = 0.140 | OS *P* = 0.013  Recurrence  *P* = 0.012 | OS *P* = 0.100  Recurrence  *P* = 0.770 | OS *P* = 0.790  Recurrence  *P* = 0.500 | OS *P* = 0.200  Recurrence  *P* = 0.860 | OS *P* = 0.008  Recurrence  *P* = 0.860 | BCKHDA and DBT show significant related to overall survival; GPR146, SELP and BCKHDA show significant related to recurrence (figure S12). |

Note: OS, Overall survival; D/PF, Disease/Progression-free; NA, not available; CGGA, the Chinese Glioma Genome Atlas.
